# The Synthesis of Novel *aza*-Steroids and *α*, *β*-Unsaturated-Cyanoketone from Diosgenin

**DOI:** 10.3390/molecules28217283

**Published:** 2023-10-26

**Authors:** Dayana Mesa, Yarelys E. Augusto, Giselle Hernández, Juan P. Figueroa-Macías, Francisco Coll, Andrés F. Olea, María Núñez, Hernán Astudillo Campo, Yamilet Coll, Luis Espinoza

**Affiliations:** 1 Center for Natural Product Researches, Faculty of Chemistry, University of Havana, Zapata and G, Vedado, Havana 10400, Cuba; dayana.mesa@fq.uh.cu (D.M.); syleray91@gmail.com (Y.E.A.); giselle_hernandez@fq.uh.cu (G.H.); juan.figueroa@fq.uh.cu (J.P.F.-M.); colladofm41@gmail.com (F.C.); 2 Grupo QBAB, Facultad de Ingeniería, Instituto de Ciencias Químicas Aplicadas, Universidad Autónoma de Chile, Llano Subercaseaux 2801, Santiago 7500912, Chile; andres.olea@uautonoma.cl; 3 Departamento de Química, Universidad Técnica Federico Santa María, Av. España No. 1680, Valparaíso 2390123, Chile; maria.nunezg@usm.cl; 4 Grupo de Investigación en Procesos Electroquímicos, Departamento de Química, Universidad del Cauca, Calle 5 No. 4-70, Popayán 190003, Colombia

**Keywords:** steroids, steroidal oximes, spirostanic oximes

## Abstract

Recent studies have demonstrated the antiproliferative and cytotoxic effects of aza-steroids and steroidal sapogenins on human cancer cell lines. The scientific community has shown a growing interest in these compounds as drug candidates for cancer treatment. In the current work, we report the synthesis of new diosgenin oxime derivatives as potential antiproliferative agents. From (25 *R*)-5α-spirost-3,5,6-triol (**1**), a diosgenin derivative, ketones **2**, **3**, **4,** and **9** were obtained and used as precursors of the new oximes. A condensation reaction was carried out between the steroidal ketones (**2**, **3**, **4**, and **9**) with hydroxylamine hydrochloride in 2,4,6-trimethylpyridine to produce five spirostanic oximes (four of them are not reported before) with a 42–96% yield. Also, a new spirostanic *α*, *β*-unsaturated cyanoketone was synthesized via Beckmann fragmentation using thionyl chloride with a 62% yield. Furthermore, we proposed a reaction mechanism with the aim of explaining such transformation.

## 1. Introduction

Cancer is a pathology which exhibits a high morbidity and mortality and therefore produces an enormous socioeconomic impact. Despite the scientific advances in this field, its incidence has risen in recent years [1,2]. In this context, breast cancer is one of the most active fields, because is the most common malignant neoplasm in women worldwide, and most of them are invasive and depend on estrogen for continued growth [3]. Thus, one of the most effective therapeutic approaches to treating hormone-dependent breast cancer is to deprive cancer cells of estrogens by using drugs acting on the estrogen receptor (ER) or inhibiting the aromatase enzyme [3,4,5,6]. Much effort has been dedicated to the quest of compounds that bind to ER, activating or inhibiting ERs selectively [5,7,8]. This group of compounds, called Selective Estrogen Receptor Modulators (SERMs), effectively block the activation of ERα by endogenous ligands and prevent the transcription of genes mediated by estrogen response elements. In this context, natural products emerge as a promising alternative, because several new anticancer agents have been tested and developed [9,10,11,12]. The development of new drugs based on natural products generally requires a starting metabolite, which exhibits some interesting activity and is chemically modified to enhance the initial activity of the parent compound [13]. Following this line, steroidal sapogenins are a very interesting group of natural compounds, because many of their biological activities have already been well established [14]. Diosgenin [(25R)-spirost-5-en-3β-ol] is a steroidal sapogenin that has been isolated from the seeds of Discoreatokoro fenugreek (Trigonella foenum graecum) [15], roots of wild yam (Dioskorea villosa) [16], and rhizome of Costus speciosus [17]. A series of biological activities have been described for diosgenin [18,19], and it is also used as the main precursor when obtaining synthetic steroids. Additionally, a large body of evidence has been accumulated regarding its anticancer activity [14,18,19,20,21].

On the other hand, several diosgenin derivatives have been synthesized and their antiproliferative activity has been assessed [22,23,24]. Interestingly, diosgenin-derived oximes have shown enhanced antiproliferative activity as compared to diosgenin [22,25]. Therefore, the use of diosgenin as a scaffold for the attachment of hydroximino groups to the steroidal rings A and B should lead to derivatives with increased anticancer activity. Jindal et al. [26] synthesized and evaluated several steroidal oximes as inhibitors of aromatase cytochrome P450, a key enzyme in estrogen biosynthesis that is responsible for hormone-dependent breast cancer. In addition, it has been reported that steroidal oximes can act as antiproliferative inhibitors of 5-reductases enzymes [27].

In this report, we present the synthetic routes of five diosgenin-derived oximes. To the best of our knowledge, four of them have not been reported before. We also obtained a new spirostanic *α*, *β*-unsaturated cyanoketone through the Beckmann fragmentation of compound **10**. Furthermore, we propose the mechanism of this reaction to explain such transformation.

## 2. Results and Discussion

### 2.1. Synthesis

**Scheme 1:**
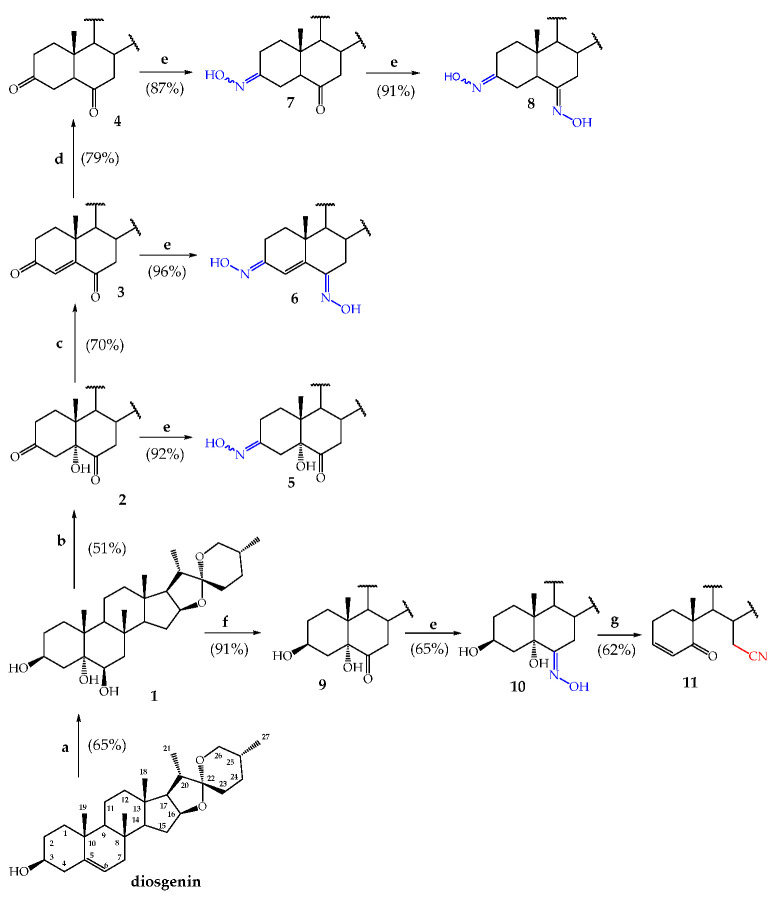
Synthesis of steroidal oximes and *α*, *β*-unsaturated-cyanoketone. Reagents and conditions: (a) NBS/(CH_3_)_2_CO, H_2_O/CH_3_CO_2_H. (b) Jones reagent (CrO_3_/H_2_SO_4_/H_2_O)/2-butanone. (c) 1. Jones reagent, 2. H_2_SO_4_ (5 mL, 50%). (d) HI/acetone. (e) NH_2_OH·HCl/ TMP. (f) NBS aq. dioxane. (g) SOCl_2_ /THF.

The introduction of a hydroxyimino group on different positions of the steroidal framework of diosgenin has been described by various groups [22]. Our strategy for the synthesis of hydroxyamino derivatives of diosgenin was to react steroidal ketones **2**, **3**, **4,** and **9** with hydroxylamine hydrochloride (NH_2_OH·HCl), as described in Scheme 1. It has been reported that a 5:6 double bond of diosgenin can be easily hydroxylated by a reaction with *N*-bromosuccinimide (NBS), leading to the trans glycolic, 5α,6β-diol (**1**). A subsequent reaction of **1** with NBS gives compound **9** [28]. Thus, the triol **1** and steroidal ketone **9** were obtained from diosgenin with high yields.

Steroidal ketone **2** was synthesized from **1** through the oxidation of secondary hydroxyl groups with Jones reagent in the presence of sulfuric acid, following a previously reported procedure [29]. In the same way, ketone **3** was obtained from **1** using the same reaction described to obtain compound **2**, but by adding concentrated sulfuric acid after all the starting triol had been consumed. Finally, ketone **4** was obtained from **3** via a regioselective reduction of the double bond with HI, at a 79% yield [30]. All the steroidal ketones were characterized using ^1^H and ^13^C NMR spectroscopies (Figures S1–S4, Supplementary Material). The data for compounds **2** and **3** are consistent with previously reported data (Figures S2 and S3, Supplementary Material). For ketone **4,** the ^13^C-NMR spectra showed two signals at δc = 211.1 ppm (C-3) and 208.8 ppm (C-6), which confirmed that carbonyl groups were not transformed under the reaction conditions. Also, the signals of the allylic carbons at C-4 and C-5 were absent, and instead new signals appeared at δc = 37.1 ppm (C-4) and 57.6 ppm (C-5), as evidence of the hydrogenation of the double bond. ^1^H-NMR showed the characteristic signals of a non-modified spiro-ketalic system (H-16, δ_H_ = 4.41 ppm and H-26 (eq /ax): δ_H_ = 3.46/3.35 ppm).

#### Synthesis of Steroidal Oximes

The oximes were prepared through a simple mild condensation reaction of steroidal ketones with NH_2_OH·HCl and 2,4,6-trimethylpyridine (TMP) as a solvent [31,32]. The reaction was monitored using TLC until the starting material was totally consumed.

A comparison of the ^13^C-NMR spectra obtained for the precursor ketones **2**, **3,** and **4** and the reaction products allows identification of the steroidal oximes **5**, **6**, **7,** and **8** (Figures S5–S8, Supplementary Material). The main signals used in this analysis are listed in Table 1. For example, in the ^13^C-NMR spectra of the starting ketones, double signals appeared around δc = 211 ppm, which are assigned to the carbonyl groups at C-3 and C-6. On the other hand, in oxime formation, these carbonyl groups were changed by hydroxyamino groups. Consequently, these C atoms gave new signals around δc = 159 ppm, instead of those due to the carbonyl groups. In addition, the presence of hydroxymino groups affected the signals of the C atoms that were bound to C-3 and C-6. Thus, the signals of C-2 and C-4 were shifted by the formation of this group at C-3, and the signal of C-7 was affected by the formation of this group at C-6.

The reaction of ketone **2** can be monitored by following the signals of C-3 and C-6 at δc = 211 ppm, and the reaction was considered to be completed when one of these signals disappeared, giving rise to new signals around δc = 159 ppm. As the signals of C-6 and C-7 remained unaltered, whereas signals C-3, C-2, and C-4 were shifted, it can be concluded that just one hydroxyamino group reacted by attachment to C-3. In other words, the data in Table 1 indicate that hydroxylamine was preferentially attached to C-3, exclusively giving oxime **5**. Interestingly, signals arising from C-3, C-2, and C-4 in oxime **5** were detected as very closed pairs, suggesting that the product is a mixture of *E*/*Z* isomers, with a 92% yield.

Similar results were obtained in the reaction of ketone **4** with hydroxylamine. Following the previously described procedure (Scheme 1), compound **7**, a mono-oxime at C-3, was obtained as a single product. However, under the same conditions, but with increasing the reaction time (from 2 to 3 h), dioxime **8** was obtained with a 91% yield. The structural characterization of oximes **7** and **8** was carried out using spectroscopic techniques. In the ^13^C-NMR spectrum of compound **7**, signals at δc = 209 and 159 ppm appeared, corresponding to the carbonyl and oxime groups at C-6 and C-3, respectively. Additionally, the signals of oxime neighbors C atoms (C-2 and C-4) were also shifted up-field (Table 1). On the other hand, a comparison of the ^13^C-NMR spectra of dioxime **8** and ketone **4** indicates the presence of oxime groups at C-3 and C-6 (signals around δc = 159 ppm). Double signals at C-2 and C-4 were also observed and corresponded to the same kind of interaction of these C atoms with the oxime group at C-3 discussed for compound **7**. However, C-7 gave a single signal shifted up-field from δc = 48.4 ppm to δc = 29.5 ppm. In summary, these results suggest that compound **7** is a mono oxime formed at C-3 and there is a mixture of the *E* and *Z* isomers, whereas compound **8** is dioxime with hydroxyamino groups at C-3 and C-6, but a mixture of isomers was observed only for the oxime formed at C-3. The oxime at C-6 had only one configuration.

Using ^1^H-NMR and two-dimensional spectra, it was possible to identify the nature of the *E* and *Z* isomers mixture of oximes **7** and **8** (Figures S7 and S8, Supplementary Material). Krstić et al., used ^1^H-NMR to identify the *E* and *Z* stereoisomers of oximes [33]. Briefly, it was shown that, in a configuration where the hydroxyl oxygen of the oxime is closer to the equatorial H from the alpha carbon, the signal of this H will be shifted to higher fields as compared to the signal observed for the starting material. In Table 2, the results of the ^1^H-NMR and two-dimensional spectra obtained for **7** and **8** are compared with the precursor ketone **4**.

The data in Table 2 show that, in oxime **7,** two different signals were observed for H-2_eq_. By comparison with this signal in compound **4** (δ_H_ = 2.11 ppm), one signal was unshielded (δ_H_ = 3.27 ppm) and the other was slightly shifted to lower fields (δ_H_ = 1.98) confirming the existence of (*E*) and (Z) stereoisomers of **7**, respectively. In oxime **8**, the same behavior was found for the H-2_eq_ signal, indicating that there were *E* and *Z* isomers for the oxime group at C-3. Interestingly, H-7eq gave just one signal at a higher field as compared to the starting material (δ_H_ = 3.35 ppm), indicating that the C-6 oxime had only one *E* configuration. Therefore, the ^1^H-NMR data confirm that compound **7** is a mono oxime formed at C-3 as a mixture of *E* and *Z* isomers. On the other hand, compound **8** is a dioxime with oxime groups at C-3 and C-6, with isomers *E* and *Z* at C-3, and only one *E* isomer at C-6.

Compound **6** was synthesized from ketone **3** under the same conditions as those used for the synthesis of **5**. The reaction time was increased to 8 h to ensure the formation of a dioxime at C-3 and C-6. The presence of only one spot in the chromatographic plate confirmed the obtention of **6** with a very high yield. The ^13^C-NMR spectrum of compound **6** showed signals at δc = 156.9 ppm, corresponding oxime groups at C-3 and C-6, respectively. The C-2 signal was shifted to stronger fields, suggesting a Z configuration of the oxime group at C-3. On the other hand, the C-7 signal was unshielded, indicating an *E* configuration for this oxime, which will be also responsible of the unshielded signal of C-4 (Figure S6, Supplementary Material). Compound **6** has been synthesized previously following a different synthetic route, and there is total agreement between the spectroscopy data reported herein and those found in the literature [22].

The reaction of compound **9** with hydroxylamine led to oxime **10** after 6 h with a 65% yield (Scheme 1). In the ^13^C-NMR spectrum, a signal of C-6 appeared at δc = 161.1 ppm, indicating that the carbonyl group of the starting compound **9** was totally converted into the oxime (Figure S9, Supplementary Material). The signals of C-3 and C-5, which were bonded to hydroxyl groups, appeared at δc = 66.5 and δc = 76.1 ppm, respectively. The configuration of oxime **10** was corroborated by an analysis of ^1^H-NMR, more specifically following the shift of the H-7_eq_ signal. Spectral correlations between the signals of C-6 and H-7_eq_ were demonstrated by HMBC spectra (Figure S10, Supplementary Material). As discussed above, it has been reported that, for the *E-* isomer in the B ring of steroidal oximes, H-7_eq_ shifts to downfield around δ_H_ = 3.00 ppm, while for the *Z*-isomer, this signal shifts to roughly δ_H_ = 2.40 ppm [33]. In the ^1^H-NMR spectra, a signal (dd) corresponding to H-7_eq_ appeared around δ_H_ = 2.9 ppm. In comparison with the parent compound **9,** this signal was shifted downfield, indicating that oxime **10** is the *E*-isomer.

Thus, our results suggest that the formation of oximes via the reaction of hydroxylamine with steroidal ketones occurs in two stages, i.e., first, the hydroxyimino groups are formed preferentially at the three-position of the steroid, and after that, all carbonyl groups at that position are transformed into oximes, and hydroxylamine starts reacting with the carbonyl groups at C-6. In compound **2,** this difference in reactivity could be attributed to the lowest reactivity of C-6 to nucleophilic attack due to the presence of the hydroxyl in the α-C. This conclusion is corroborated by the lowest reactivity of compound **9**, in which the -OH and -CO groups were also located at C-5 and C-6, respectively.

In addition, the analysis of the NMR spectroscopic data obtained for oximes **6**, **7**, and **8** indicated that the formation of oxime at C-6 was stereospecific, since, in all these oximes, the hydroxyimino group adopted the *E* configuration. The steric hindrances presented by the carbonyl group in that position make it possible to force the regioselectivity or stereoselectivity of the chemical reaction or minimize unwanted side reactions.

These experimental facts are explained in the iminium ion formation step, which can adopt *E* or *Z* stereochemistry.

**Figure 1:**
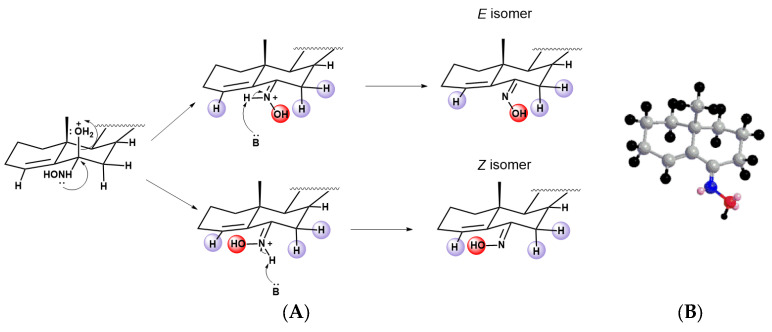
(**A**) Deprotonation step of the iminium ion formed in reaction of compound **3** and hydroxylamine leading to *E* or *Z* oxime isomers of oxime **6**; (**B**) spatial distribution of *E* and *Z* isomers of an oxime. Colored balls represent H atoms, black; C atoms, gray; N atoms, blue; O atoms, red; and electrons, pink.

In Figure 1, it is observed that the *E* ion presents lowest steric repulsions, having an additional stabilizing effect. This effect favors the formation of *E*-isomer and explains why only this isomer was formed at position 6 of the steroid.

**Figure 2:**
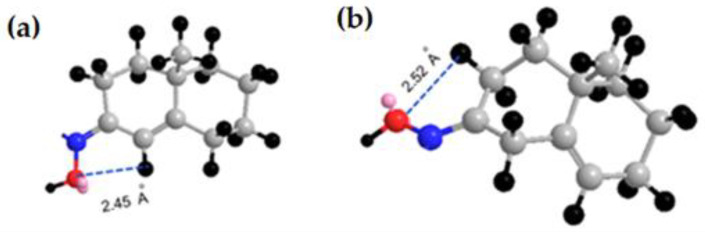
Bond distance of the hydroxyl group of the oxime to the equatorial hydrogens of the configuration (**a**) *Z* and (**b**) *E*. Colored balls represent H atoms, black; C atoms, gray; N atoms, blue; O atoms, red; and electrons, pink.

In the case of the oximes in position 3, the distances between the hydroxyl group of the oxime to the equatorial hydrogen of both position 2 and position 4 are very similar (Figure 2), and therefore no additional stabilization due to steric hindrances is obtained.

It has been reported that a group in the α position to the hydroxyimino moiety, which is able to stabilize the positive charge of the intermediate, favors Beckmann fragmentation instead of Beckmann rearrangement. This fragmentation produces a variety of compounds depending on the structure and other functionalities present in the oxime [34,35]. In general, it is commonly accepted that *syn*-α-hydroxyoximes lead to aldehydes (ketones) and isonitriles, whereas anti-isomers afford aldehydes (ketones) and nitriles. In our case, oxime **10** (anti-isomer) underwent Beckmann fragmentation in the presence of SOCl_2_ in THF at 0 °C and, surprisingly, an *α*, *β*-unsaturated-cyanoketone was obtained (**11**). The chemical structure of **11** was confirmed by the NMR spectra. An analysis of the ^1^H-NMR spectrum of **11** showed two signals around δ_H_ = 6.84 and 6.02 ppm, corresponding to the allylic protons H-3 and H-4, respectively, which were shifted downfield compared to those at the derivative **10** (H-3; δ_H_ = 4.67 ppm), whereas a correlation between H-4 and carbon C-5 was found using HMBC (see support information). On the other hand, in the ^13^C-NMR spectrum, a signal appearing at δc = 208.3 ppm confirmed the presence of *α*, *β*-unsaturated ketone, a signal around δc = 117.8 ppm was assigned to a nitrile function, and resonances at δc = 147.4 ppm and δc = 128.6 ppm corresponded to an alkene functionality.

**Scheme 2:**
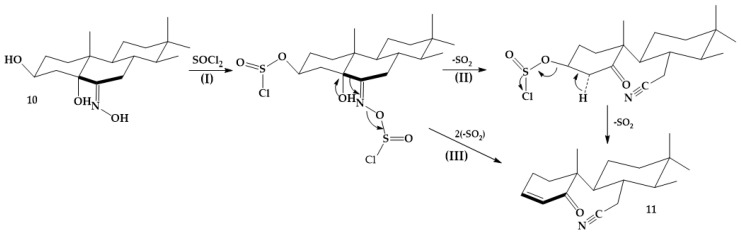
Mechanism of Beckman fragmentation induced by SOCl_2_.

The proposed mechanism of the SOCl_2_-induced Beckmann fragmentation of oxime **10**, whose hydroxyl group is *anti* to the carbon C-5, is depicted in Scheme 2.

According to this scheme, mono-chloro sulfites are produced by thionyl chloride attack on the hydroxyl group at C-3 and the hydroxyimino group at C-6 (Scheme 2, Stage I). Subsequently, the elimination of alkylchlorosulfite at C-3 could occur concertedly or sequentially with imine system decomposition to give compound **11** (Scheme 2, Stage II and III). Stereospecific fragmentation, with the bond cleavage (C_5_-C_6_) of the *E*-isomer of oxime **10,** leads to ketone and nitrile (Scheme 2, Stage III and IV). The cyclohexanone in ring A adopts a very tense semi-boat conformation, in which the H_4α_ and the alkylchlorosulfite at C-3 are in quasi-axial positions. These effects favor the elimination of H_4α_, leading to the *α*, *β*-unsaturated cyanoketone (Scheme 2).

## 3. Materials and Methods

### 3.1. General Experimental Procedures

The melting points were determined on Stuart Scientific apparatus and were uncorrected. NMR spectra (^1^H, ^13^C COSY, TOCSY, NOESY, HSQC, and HMBC) were recorded on a Varian Mercury spectrometer (400 MHz for ^1^H, 100 MHz for ^13^C) in CDCl_3_ or DMSO-d_6_ at room temperature. Chemical shifts are reported in ppm (*δ*) and coupling constants (*J*) in Hz. Spectra processing was performed using the MestReNova 16.0 software. High-resolution mass spectra (HRMS) were recorded on an Orbitrap Elite mass spectrometer (Thermo Fisher Scientific, Bremen, Germany) equipped with a heated ESI electrospray ion source. Thin-layer chromatography was performed on aluminum plates precoated with Merck silica gel 60 F254, detection with 50% aq. H_2_SO_4_, and were heated until color developed. Preparative column chromatography was performed on Silica gel Merck (0.063–0.200 mm). The removal of solvents was carried out under reduced pressure.

### 3.2. Synthesis of Steroidal Ketones

The starting material, compound **1**, was synthesized according to a previously reported procedure [29]. Briefly, a suspension of diosgenin (5 g, 0.012 mol) in acetone (200 mL) and water (25 mL) was treated with NBS (2.68 g, 0.15 mol) and acetic acid (2.5 mL) at room temperature. Over 45 min, the reaction mixture went from yellow to orange and finally became colorless when all the solid material had disappeared. The reaction mixture was left overnight, diluted with water, extracted with ether, and processed as usual to give an amorphous solid (5.9 g, 67% yield). Crystallization from MeOH gave white product **1**. Compound **1**, mp: 280–282 °C (MeOH), (281–283 °C ref. [29]), ^1^H NMR (400 MHz, CDCl_3_) (Figure S1, Supplementary Material): 0.76–0.67 (m, 6H, H-27 and H-18); 0.89 (d, *J* = 6.9 Hz, 3H, H-21); 1.03 (s, 3H, H-19); 3.20 (t, *J* = 11.0 Hz, 1H, H-26 ax); 3.40 (d, 1H, H-26 eq); 3.65 (s, 1H, C5-OH); 3.78 (dt, *J* = 16.2 and 5.5 Hz, 1H, H-3); 4.15 (d, *J* = 5.6 Hz, 1H, C3-OH); 4.26 (q, *J* = 7.8 Hz, 1H, H-16); 4.43 (d, *J* = 4.2 Hz, 1H, C6-OH). ^13^C NMR (100 MHz, DMSO-d6) (Figure S1, Supplementary Material): 31.5 (C-1); 30.9 (C-2); 65.9 (C-3); 40.9 (C-4); 74.3 (C-5); 74.0 (C-6); 34.7 (C-7); 31.1 (C-8); 44.6 (C-9); 37.9 (C-10); 20.5 (C-11); 38.9 (C-12); 40.2 (C-13); 55.5 (C-14); 32.0 (C-15); 80.3 (C-16); 61.9 (C-17); 16.2 (C-18); 16.3 (C-19); 41.1 (C-20); 14.6 (C-21); 108.4 (C-22); 29.8 (C-23); 28.5 (C-24); 29.6 (C-25); 65.7 (C-26); 17.1 (C-27). HRMS-ESI: 447.3585 [M − H]^−^ (calculated for: C_27_H_44_O_5_: 448.3266).

#### 3.2.1. (25*R*)-Spirost-5α-hydroxy-3,6-dione (**2**)

A freshly prepared Jones reagent (20 mL; 53.4 mmol) was added dropwise to a solution of steroidal sapogenin **1** (5 g, 11.2 mmol) in butanone (200 mL). The solution was stirred for 4 h at room temperature. The reaction was quenched with isopropanol and was then filtered. The resulting solution was washed with K_2_CO_3_ (3 × 10 mL) and brine (3 × 10 mL), dried over Na_2_SO_4_, and evaporated under reduced pressure to produce a white solid. Crystallization from hexane/EtOAc afforded the pure (25*R*)-3,6-dioxo-5α-spirost-5-ol (**2**) (2.0 g, 51% yield), mp: 282–283 °C (MeOH), (283 °C (MeOH) [30]), ^1^H NMR (400 MHz, CDCl_3_) (Figure S2, Supplementary Material): 0.72 (s, 3H, H-18); 0.73 (d, *J* = 7.8 Hz, H-27); 0.91 (bs, 6H, H-19 and H-21); 3.22 (t, *J* = 11.0 Hz, 1H, H-26 ax); 3.34–3.47 (m, 1H, H-26 eq), 4.30 (q, *J* = 7.3 Hz, 1H, H-16); 5.95 (s, 1H, OH). ^13^C NMR (100 MHz, DMSO-d_6_) (Figure S2, Supplementary Material): 31.1 (C-1); 36.8 (C-2); 210.0 (C-3); 44.2 (C-4); 80.0 (C-5); 210.7 (C-6); 41.1 (C-7); 36.5 (C-8); 44.0 (C-9); 42.7 (C-10); 20.9 (C-11); 40.2 (C-12); 40.5 (C-13); 55.4 (C-14); 30.9 (C-15); 81.4 (C-16); 61.6 (C-17); 16.1 (C-18); 13.3 (C-19); 41.5 (C-20); 14.6 (C-21); 108.4 (C-22); 31.1 (C-23); 28.5 (C-24); 29.8 (C-25); 65.9 (C-26); 17.1 (C-27). HRMS-ESI: 443.2185 [M − H]^−^ (calculated for: C_27_H_40_O_5_: 443.2876).

#### 3.2.2. (25*R*)-Spirost-4-en-3,6-dione (**3**)

A freshly prepared Jones reagent (25 mL, 66.75 mmol) was added dropwise to a solution of steroidal sapogenin **2** (5g, 11.2 mmol) in butanone (200 mL). H_2_SO_4_ (5 mL, 50%) was added and the reaction mixture was stirred until compound **2** was consumed (TLC, approx. 6 h). Isopropanol was added to this solution and the reaction mixture was filtered. The liquid was washed with K_2_CO_3_ (3 × 10 mL) and brine (3 × 10 mL), dried over Na_2_SO_4_, and evaporated under reduced pressure to produce a solid. The residue was purified using column chromatographic (hexane/EtOAc 7:1) to give pure white solid (25*R*)-spirost-4-en-3,6-dione (**3**), (3.34 g, 70% yield); mp: 190–191 °C (hexane/EtOAc), (193–194 °C [22]). ^1^H NMR (400 MHz, CDCl_3_) (Figure S3, Supplementary Material): 0.80 (d, *J* = 6.3 Hz, 3H, H-27); 0.80 (s, 3H, H-18); 0.99 (d, *J* = 6.8 H, 3H, H-21); 1.19 (s, 3H, H-19); 3.30 (t, *J* = 10.9 Hz, 1H, H-26 ax); 3.41 (dd, *J =* 10.8 Hz, 1H, H-26 eq); 4.31–4.41 (m, 1H, H-16 ax); 6.12 (s, 1H, H-4). ^13^C NMR (100 MHz, CDCl_3_) (Figure S3, Supplementary Material): 35.7 (C-1); 34.1 (C-2); 199.3 (C-3); 125.7 (C-4); 160.6 (C-5); 201.8 (C-6); 47.0 (C-7); 34.0 (C-8); 51.0 (C-9); 39.9 (C-10); 20.8 (C-11); 39.3 (C-12); 41.8 (C-13); 56.4 (C-14); 31.5 (C-15); 80.4 (C-16); 62.1 (C-17); 16.4 (C-18); 17.7 (C-19); 40.6 (C-20); 14.6 (C-21); 109.5 (C-22); 31.6 (C-23); 28.9 (C-24); 30.4 (C-25); 67.0 (C-26); 17.3 (C-27). HRMS-ESI: 425.2576 [M − H]^−^ (calculated for: C_27_H_37_O_4_: 425.2770).

#### 3.2.3. (25*R*)-5α-Spirost-3,6-dione (**4**)

To a solution of compound **3** (0.8 g, 1.9 mmol) in acetone (27.5 mL), HI (3.3 mL, 44 mmol) was added. The reaction mixture was stirred at room temperature until the starting material was consumed (TLC, approx. 2 h). Aqueous saturated solutions of NaHCO_3_ (5 mL) and Na_2_S_2_O_3_ (solid) were added until the solution color faded. The reaction mixture was filtered. The liquid and solid were washed with DCM (3 × 10 mL). The organic phase was dried over Na_2_SO_4_ and evaporated under reduced pressure to produce a pale yellow solid that was recrystallized in acetone to afford (25 *R*)-5α-spirost-3,6-dione (**4**), (633 mg, 79% yield), mp: 226–227 °C (acetone). ^1^H NMR (400 MHz, CDCl_3_) (Figure S4, Supplementary Material): 0.79 (d, *J* = 6.8 Hz, H-27); 0.81 (s, 3H, H-18); 0.98 (s, 3H, H-19); 0.98 (d, *J* = 6.5 Hz, H-21); 2.29–2.38 (m, 1H, H-4 ax); 2.38–2.42 (m, 1H, H-5); 2.56–2.62 (m, 1H, H-4 eq); 3.35 (t, *J* = 10.9 Hz, 1H, H-26 ax); 3.46 (ddd, *J* = 10.8, 4.6 and 2.0 Hz, 1H, H-26 eq); 4.41 (td, *J* = 7.8, 7.3 and 5.9 Hz, 1H, H-16α). ^13^C NMR (100 MHz, CDCl_3_) (Figure S4, Supplementary Material): 38.2 (C-1); 37.5 (C-2); 211.1 (C-3); 37.1 (C-4); 57.6 (C-5); 208.8 (C-6); 46.8 (C-7); 37.6 (C-8); 53.6 (C-9); 41.1 (C-10); 21.7 (C-11); 39.5 (C-12); 41.3 (C-13); 56.5 (C-14); 31.5 (C-15); 80.5 (C-16); 62.2 (C-17); 16.5 (C-18); 12.8 (C-19); 41.8 (C-20); 14.6 (C-21); 109.4 (C-22); 31.7 (C-23); 28.9 (C-24); 30.4 (C-25); 67.0 (C-26); 17.3 (C-27). HRMS-ESI: 429.3005 [M + H]^+^ (calculated for: C_27_H_41_O_4_: 429.2927).

### 3.3. General Procedure for the Synthesis of Steroidal Oximes

To a solution of keto-steroid (0.2 mg, 0.45 mmol) in 2 mL of 2,4,6-trimethylpyridine (TMP), NH_2_OH.HCl (65.2 mg, 0.93 mmol) dissolved in 2 mL of TMP was added. The reaction mixture was stirred at room temperature until the starting material was consumed (TLC, see Table 3 for reaction times and yields). The reaction product was poured into water and the solid was filtered and washed with 5% aq HCl.

#### 3.3.1. (25*R*)-(3*E*/*Z*)-Hydroximino-5α-spirost-5-hydroxy-6-ona (**5**)

Following the procedure described in Section 3.3 a crude product was obtained after 6 h. This residue was purified using column chromatography (hexane/EtOAc 1:1) to give a white solid mixture of the *E* and *Z* isomers of compound **5** (218 mg, 92% overall yield). ^1^H NMR (400 MHz, CDCl_3_) (Figure S5, Supplementary Material): 0.76 (s, 3H, H-18); 0.79 (d, *J* = 6.3 Hz, H-27); 0.89 (s, 3H, H-19); 0.97 (d, *J* = 6.7 Hz, 3H, H-21); 3.31–3.19 (m, 1H, H-2 eq (**5** (*E*)) and H-4 eq (**5** (*Z*)); 3.36 (t, *J* = 10.9 Hz, 1H, H-26 ax.); 3.48 (tdd, *J* = 10.1, 9.6 and 4.1 Hz, 1H, H-26 eq); 4.25–4.52 (m, 1H, H-16). ^13^C-NMR (100 MHz, CDCl_3_) (Figure S5, Supplementary Material): 31.7 (C-1); 20.1 (C-2, isomers *E*); 25.7 (C-2, isomers *Z*); 159.6 (C-3, isomers *E*); 159.2 (C-3, isomers *Z*); 29.8 (C-4, isomers *E*); 27.7 (C-4, isomers *Z*); 80.3 (C-5, isomers *E*); 80.4 (C-5, isomers *Z*); 212.0 (C-6, isomers *E*); 211.6 (C-6, isomers *Z*); 42.2 (C-7, isomers *E*); 42.4 (C-7, isomers *Z*); 36.7 (C-8, isomers *E*); 36.9 (C-8, isomers *Z*); 44.7 (C-9); 43.5 (C-10, isomers *E*); 43.8 (C-10, isomers *Z*); 21.2 (C-11); 39.6 (C-12); 41.1 (C-13); 56.1 (C-14); 33.9 (C-15); 80.6 (C-16); 62.2 (C-17); 16.5 (C-18); 13.8 (C-19); 41.8 (C-20); 14.6 (C-21);109.4 (C-22); 31.5 (C-23); 28.7 (C-24); 30.4 (C-25); 67.0 (C-26); 17.3 (C-27). HRMS-ESI: 460.2616 [M + H]^+^ (calculated for: C_27_H_42_NO_5_: 460.2985).

#### 3.3.2. (25*R*)-(3*Z*,6*E*)-Dihydroximinospirost-4-ene (**6**)

Using the procedure described in Section 3.3, a crude product was obtained after 8 h. Recrystallization from hexane/EtOAc gave a white solid **6**, (206 mg, 96% yield), mp: 149–151 °C (hexane/EtOAc), (150–152 °C, [22]). ^1^H NMR (400 MHz, CDCl_3_) (Figure S6, Supplementary Material): 0.79 (d, *J* = 6.8 Hz, 3H, H-27); 0.80 (s, 3H, H-18); 0.98 (d, *J* = 6.9 Hz, 3H, H-21); 1.02 (s, 3H, H-19); 3.35–3.42 (m, 1H, H-26 ax.); 3.47 (dd, *J* = 11.3 and 4.3 Hz, 1H, H-26 eq); 4.42 (q, *J* = 7.5 Hz, 1H, H-16); 6.48 (s, 1H, H-4). ^13^C NMR (100 MHz, CDCl_3_) (Figure S6, Supplementary Material): 18.7 (C-1); 31.5 (C-2); 156.8 (C-3); 119.4 (C-4); 147.7 (C-5); 156.9 (C-6); 29.9 (C-7); 32.7 (C-8); 51.3 (C-9); 38.4 (C-10); 21.2 (C-11); 33.7 (C 12); 40.6 (C-13); 56.6 (C-14); 31.8 (C-15); 80.8 (C-16); 62.1 (C-17); 16.5 (C-18); 17.7 (C-19); 41.8 (C-20); 14.6 (C-21); 109.5 (C-22); 39.6 (C-23); 28.9 (C-24); 30.4 (C-25); 67.0 (C-26); 17.3 (C-27). HRMS-ESI: 456.2982 [M + H]^+^, (calculated for: C_27_H_40_O_4_N_2_: 456.2988).

#### 3.3.3. (25*R*)-(3*E*/*Z*)-Hydroximino-5α-spirost-6-ona (**7**)

The procedure described in Section 3.3, after 2 h, gave a crude product. Recrystallization from acetone afforded the mixture of the *E* and *Z* isomers of compound **7** (175 mg, 87% yield). ^1^H NMR (400 MHz, CDCl_3_) (Figure S7, Supplementary Material): 0.79 (s, 3H, H-18); 0.79 (d, *J* = 6.3 Hz, H-27); 0.87 (s, 3H, H-19); 0.98 (d, *J* = 6.8 Hz, 3H, H-21); 3.28 (ddt, *J* = 15.6, 4.8 and 2.1 Hz, 2H, H-2 eq (**7**(*E*)) and H-4 eq (**7**(*Z*)) 3.36 (t, *J* = 10.9 Hz, 1H, H-26 ax.); 3.47 (ddd, *J* = 11.0, 4.6 and 2.0 Hz, H-26 eq); 4.44–4.39 (m, 1H, H-16); 7.93–7.52 (bs, 2H, OH). ^13^C NMR (100 MHz, CDCl_3_) (Figure S7, Supplementary Material): 37.0 and 38.2 (C-1, isomers *E* and *Z*); 19.8 (C-2, isomers *E*); 26.9 (C-2, isomers *Z*); 159.9 and 159.1 (C-3, isomers *E* and *Z*); 19.9 (C-4, isomers Z); 27.1 (C-4, isomers *E*); 56.5 and 57.7 (C-5, isomers *E* and *Z*); 209.7 and 209.8 (C-6 isomers *E* and *Z*); 46.6 (C-7); 37.3 (C-8); 53.5 (C-9); 40.8 (C-10); 21.2 (C-11); 39.3 (C-12); 41.7 (C-13); 56.2 (C-14); 31.4 (C-15); 80.2 (C-16); 61.9 (C-17); 16.2 (C-18); 112.4 (C-19); 41.5 (C-20); 14.3 (C-21); 109.2 (C-22); 31.2 (C-23); 28.5 (C-24); 30.1 (C-25); 66.7 (C-26); 16.9 (C-27). HRMS-ESI:490.2196 [M + COOH]^+^, (calculated for: C_28_H_41_NO_6_: 490.3145).

#### 3.3.4. (25*R*)-(3*E*/*Z*,6*E*)-dihydroximino-5α-spirostane (**8**)

The procedure described in Section 3.3, gave, after 3 h, a crude product. Recrystallization from hexane/EtOAc gave a white solid as a mixture of **8** isomers (194 mg, 91% overall yield). ^1^H NMR (400 MHz, CDCl_3_) (Figure S8, Supplementary Material): 0.77 (s, 3H, H-18); 0.79 (d, *J* = 6.3 Hz, 3H, H-27); 0.86 (s, 3H, H-19); 0.97 (d, *J* = 6.7 Hz, 3H, H-21); 2.47 (dt, *J* = 14.7 and 3.1 Hz, 1H, H-4eq (**8**(*E*)); 3.31–3.22 (m, 1H, H-2 eq (**8**(E)); 3.43–3.32 (m, 3H, H-26 ax, H-4 eq (**8**(*Z*)) and H-7 eq); 3.53–3.43 (m, 1H, H-26 eq); 4.42 (q, *J* = 7.0 Hz, 1H, H-16). ^13^C NMR (100 MHz, CDCl_3_) (Figure S8, Supplementary Material): 36.5 and 37.6 (C-1, isomers *E* and *Z*); 20.0(C-2, isomer *E*); 21.2 (C-2, isomer Z); 159.8 and 160.0 (C-3, isomers *E* and *Z*); 21.1 (C-4, isomer Z); 28.3 (C-4, isomer E); 49.6 (C-5, isomer *E*); 50.9 (C-5, isomer *Z*); 158.8 and 159.1 (C-6, isomers *E* and *Z*); 29.5 and 29.6 (C-7, isomer *E* and *Z*); 35.1 and 35.2 (C-8, isomers E and Z); 53.9 and 54.0 (C-9, isomers E and Z); 39.5 (C-10); 19.9 (C-11); 39.7 (C-12); 40.7 (C-13); 56.3 (C-14); 31.6 (C-15); 80.6 (C-16); 62.0 (C-17); 16.5 (C-18); 11.9 (C-19); 41.6 (C-20); 14.5 (C-21);109.3 (C-22); 33.3 (C-23); 28.7 (C-24); 30.3 (C-25); 66.9 (C-26); 17.1 (C-27). HRMS-ESI: 460.2616 [M + H]^+^, (calculated for: C_27_H_42_NO_5_: 460.2985).

#### 3.3.5. (25*R*)-(6*E*)-Hydroximino-5α-spirost-3β,5-diol (**10**)

The procedure described in Section 3.3, after 6 h, gave a crude product. Recrystallization from acetone afforded dihydroxy-oxime **10**, (134 mg, 65% yield), mp: 186–188 °C (acetone). ^1^H NMR (400 MHz, CDCl_3_) (Figure S10, Supplementary Material): 0.72 (s, 3H, H-18); 0.74 (s, 3H, H-19); 0.75 (d, *J* = 6.7 Hz, 3H, H-27); 0.92 (d, *J* = 6.9 Hz, 3H, H-21); 2.94 (dd, *J* = 13.3 and 4.5 Hz, 1H, H-7eq) 3.25 (t, *J* = 11.0 Hz, 1H, H-26 ax.); 3.42 (dd, *J* = 11.0 and 4.4 Hz, 1H, H-26 eq); 4.41–4.46 (m, 1H, H-16); 4.66–4.68 (m, 1H, H-3α); 10.4 (s, 1H, =N-OH). ^13^C NMR (100 MHz, DMSO-d_6_) (Figure S10, Supplementary Material): 27.2 (C-1); 30.1 (C-2); 66.5 (C-3); 35.0 (C-4); 76.1 (C-5); 160.2 (C-6); 24.7 (C-7); 34.6 (C-8); 43.2 (C-9); 41.6 (C-10); 21.3 (C-11); 40.9 (C-12); 41.1 (C-13); 56.1 (C-14); 31.7 (C-15); 80.8 (C-16); 62.4 (C-17); 16.8 (C-18); 16.5 (C-19); 44.9 (C-20); 14.8 (C-21); 109.0 (C-22); 31.0 (C-23); 28.9 (C-24); 30.5 (C-25); 66.1 (C-26); 17.3 (C-27). HRMS-ESI: 462.3221 [M + H]^+^ (calculated for: C_27_H_43_NO_5_: 461.3141).

#### 3.3.6. (25*R*)-5-Oxo-5,6-secospirost-3-en-6-nitrile (**11**)

To a solution of oxime **10** (0.10 g, 0.22 mmol) in THF (4 mL), a mixture of SOCl_2_ (0.46 mL) and THF (1.32 mL) was added dropwise. After the addition was concluded, stirring was continued under a nitrogen atmosphere at 0 °C, until the total consumption of the starting material. At this point, water was added and the reaction product was neutralized with NH_3_, which gave a solid. The solid was filtered, dried in vacuo, and recrystallized in acetone. Pure (25 *R*)-5-oxo-5,6-secospirost-3-en-6-nitrile (**11**) was obtained (57.3 mg, 62% yield), m.p.: 201–205 °C (acetone). ^1^H NMR (400 MHz, CDCl_3_) (Figure S11, Supplementary Material): 0.79 (d, *J* = 7.4 Hz, 3H, H-27); 0.80 (s, 3H, H-18); 0.97 (d, *J* = 6.8 Hz, 3H, H-21); 1.08 (s, 3H, H-19); 2.77 (dd, *J* = 17.8 and 3.9 Hz, 1H, H-7 ax); 2.35–2.50 (m, 1H, H-7 eq); 3.34 (t, *J* = 10.9 Hz, 1H, H-26 ax.); 3.46 (dd, *J* = 10.9 and 4.6 Hz, 1H, H-26 eq); 4.40–4.45 (m, 1H, H-16); 6.02–6.09 (m, 1H, H-4); 6.84 (ddd, *J* = 11.5, 5.0 and 2.2 Hz, 1H, H-3). ^13^C NMR (100 MHz, CDCl_3_) (Figure S11, Supplementary Material): 35.4 (C-1); 23.1 (C-2); 147.4 (C-3); 128.6 (C-4); 208.3 (C-5); 117.8 (C-6); 19.6 (C-7); 34.5 (C-8); 41.7 (C-9); 47.4 (C-10); 24.1 (C-11); 39.2 (C-12); 40.1 (C-13); 53.1 (C-14); 31.9 (C-15); 79.6 (C-16); 62.1 (C-17); 17.0 (C-18); 17.3 (C-19); 41.2 (C-20); 14.3 (C-21); 108.9 (C-22); 31.2 (C-23); 28.6 (C-24); 30.1 (C-25); 66.7 (C-26); 16.0 (C-27). DEPT-135° (*δ*, ppm): 147.5 (+); 128.7 (+); 79.8 (+); 66.88 (−); 62.2 (+); 53.3 (+); 41.8 (+); 41.3 (+); 39.3 (−); 35.5 (−); 34.6 (+); 32.0 (−); 31.4 (−); 30.2 (+); 28.8 (−); 24.3 (−); 23.3 (−); 19.8 (−); 17.4 (+); 17.1 (+); 16.2 (+); 14.5 (+). HRMS-ESI:426.3221 [M + H]^+^ (calculated for: C_27_H_40_NO_3_: 426.2930).

## 4. Conclusions

A synthetic pathway for introducing oxime groups on the steroidal A/B rings of (25 *R*)-5α-spirost-3,5,6-triol (**1**), a diosgenin derivative, was described. Steroidal ketones (**2**, **3**, **4,** and **9**) were obtained and, via condensation reaction with NH_2_OH·HCl, three mono oximes (**5**, **7**, and **10**) and two dioximes (**6** and **8**) were synthesized. Interestingly, the NMR spectroscopic characterization of these compounds indicated that the oxime formation at C-6 was stereospecific, i.e., it led only to the *E* isomer, whereas the reaction at C-3 gave both the *E* and *Z* isomers. Consequently, the *E* and *Z* isomers of oximes **5**, **7,** and **8** were identified, while only the *E* configuration of oximes **6** and **10** was observed. The regioselectivity of this reaction at C-6 was attributed to lowest steric hindrance at this position.

In addition, a new *α*, *β*-unsaturated cyanoketone (**11**) was obtained via the Beckmann fragmentation of oxime **10**.

It is worth mentioning that only oxime **6** has been previously reported. Studies of in vitro activities are in progress.

## Figures and Tables

**Table 1:** ^13^C chemical shifts (ppm) for some C atoms in precursors **2**, **3,** and **4** and products **6**, *E*/*Z* isomers of **5** and **8**.

Compound		C-3	C-6	C-2	C-4	C-7
**2**		210.0	210.7	36.8	44.2	41.1
**5**	E	159.6	212.0	20.1	29.8	42.2
	Z	159.2	211.6	25.7	27.7	42.4
**3**		199.3	201.8	34.1	125.7	47.0
**6**	*E*	156.8	156.9	31.5	119.4	29.9
**4**		211.1	208.8	37.5	37.1	48.4
**7**	*E*	159.9	209.7	19.8	27.1	46.6
	*Z*	158.1	209.8	26.9	19.9	46.6
**8**	*E*	159.8	158.8	20.0	28.3	29.5
	*Z*	160.0	159.1	21.2	21.1	29.6

**Table 2:** Selected 1H chemical shifts (ppm) for oximes **7** (*E* and *Z* b) and **8** (*E/E* and *E/Z*) and their precursor 4.

H-2_eq_		H-2_eq_	H-7_eq_
**4**		2.11	2.41
**7**	*E*	3.27	2.38
	*Z*	1.98	2.38
**8**	*E/E*	3.33	3.35
	*Z/E*	1.57	3.35

**Table 3:** Yields and reaction times for steroidal oximes (**5–8** and **10**).

Entry	Starting Material	Products	Yields (%)	Reaction Times (h)
**1**	**2**	**5**	92 ^a^	6
**2**	**3**	**6**	96 ^b^	8
**3**	**4**	**7**	87 ^b^	2
**4**		**8**	91 ^a^	3
**5**	**9**	**10**	65 ^b^	6

^a^ Yields after purification in chromatographic column. ^b^ Yield after recrystallization.

## Data Availability

The data presented in this study are available in Supplementary Material.

## Supplementary Materials

The following supporting information can be downloaded at: https://www.mdpi.com/article/10.3390/molecules28217283/s1, Figure S1: NMR spectra of 5*α*,25*R*-spirostane-3β,5,6β-triol (**1**), Figure S2: NMR spectra of (25*R*)-3,6-dioxo-5α-spirost-5-ol (**2**), Figure S3: NMR spectra of (25*R*)-20 spirost-4 –en-3,6-dione (**3**), Figure S4: NMR spectra (25*R*)-5α-spirost-3,6-dione (**4**), Figure S5: NMR spectra of (25*R*)- (3*E*/Z)-21 hydroximino-5α-spirost-5-hydroxy-6-ona (**5**), Figure S6: NMR spectra of (25R)- (3*E*,6*E*)-dihydroximinospirost-4-ene (**6**), Figure S7: NMR spectra of mixture (25*R*)-(3*E*/*Z*)-hydroximino-5α-spirost-6-ona (**7**), Figure S8: NMR spectra of mixture (25*R*)-(3*E*/*Z*,6*E*)-23 dihydroximino-5α-spirostane (**8**), Figure S9: NMR spectra of (25*R*)-3β,5α-dihydroxy-spirostan-6-ona (**9**) Figure S10: NMR spectra 24 of (25*R*)-(6*E*)-hydroximino-5α-spirost-3β,5-diol (**10**), Figure S11: NMR spectra of (25*R*)-5-oxo-5,6-secospirost-3-en-6-nitrile (**11**).
