# Supplementary material for: The Synthesis of Novel aza-Steroids and α, β-Unsaturated-Cyanoketone from Diosgenin
Source: Molecules. 2023 Oct 26;28(21):7283. doi: 10.3390/molecules28217283 (PMC10649921; doi:10.3390/molecules28217283)
Supplement: Supplementary file 1 [file molecules-28-07283-s001.zip › molecules-2654923-supplementary.pdf]

molecules

MDPI

Article

The Synthesis of Novel *aza*-Steroids and *α*, *β*-Unsaturated-Cyanoketone from Diosgenin

Dayana Mesa [1], Yarelys E. Augusto [1], Giselle Hernández [1], Juan P. Figueroa-Macías [1], Francisco Coll [1], Andrés F. Olea [2], María Núñez [3], Hernán Astudillo Campo [4], Yamilet Coll [1,*] and Luis Espinoza [3,*]

[1] Center for Natural Product Researches, Faculty of Chemistry, University of Havana, Zapata and G, Vedado, Havana 10400, Cuba; dayana.mesa@fq.uh.cu (D.M.); syleray91@gmail.com (Y.E.A.); giselle_hernandez@fq.uh.cu (G.H.); juan.figueroa@fq.uh.cu (J.P.F.-M.); colladofm41@gmail.com (F.C.)
[2] Grupo QBAB, Instituto de Ciencias Químicas Aplicadas, Facultad de Ingeniería, Universidad Autónoma de Chile, Llano Subercaseaux 2801, Santiago 7500912, Chile; andres.olea@uautonoma.cl
[3] Departamento de Química, Universidad Técnica Federico Santa María, Av. España No. 1680, Valparaíso 2390123, Chile; maria.nunezg@usm.cl
[4] Grupo de Investigación en Procesos Electroquímicos, Departamento de Química, Universidad del Cauca, Calle 5 No. 4-70, Popayán 190003, Colombia; hernanastudillo@unicauca.edu.co
* Correspondence: yamcoll@fq.uh.cu (Y.C.); luis.espinozac@usm.cl (L.E.); Tel.: +53-78792331 (Y.C.); +56-32-2654225 (L.E.)

Supplementary Materials: **The following are available online at www.mdpi.com/xxx/s1, Figure S1**: NMR spectra of *5α,25R-spirostane-3β,5,6β-triol* (**1**), **Figure S2**: NMR spectra of *(25R)-3,6-dioxo-5α-spirost-5-ol* (**2**), **Figure S3**: NMR spectra of *(25R)-spirost-4 –en-3,6-dione* (**3**), **Figure S4**: NMR spectra *(25R)- 5α-spirost-3,6-dione* (**4**), **Figure S5**: NMR spectra of *(25R)- (3E/Z)-hydroximino-5α-spirost-5-hydroxy-6-ona* (**5**), **Figure S6**: NMR spectra of *(25R)- (3E,6E)-dihydroximinospirost-4-ene* (**6**), **Figure S7**: NMR spectra of mixture *(25R)- (3E/Z)-hydroximino-5α-spirost-6-ona* (**7**), **Figure S8**: NMR spectra of mixture *(25R)- (3E/Z,6E)-dihydroximino-5α-spirostane* (**8**), **Figure S9**: NMR spectra of *(25R)-3β,5α-dihydroxy-spirostan-6-ona* (**9**) **Figure S10**: NMR spectra of *(25R)- (6E)-hydroximino-5α-spirost-3β,5-diol* (**10**), **Figure S11**: NMR spectra of *(25R)-5-oxo-5.6-secospirost-3-en-6-nitrile* (**11**).

Figure S1: NMR spectra of 5α,25*R*-spirostane-3β,5,6β-triol (**1**)

^{1}H-NMR

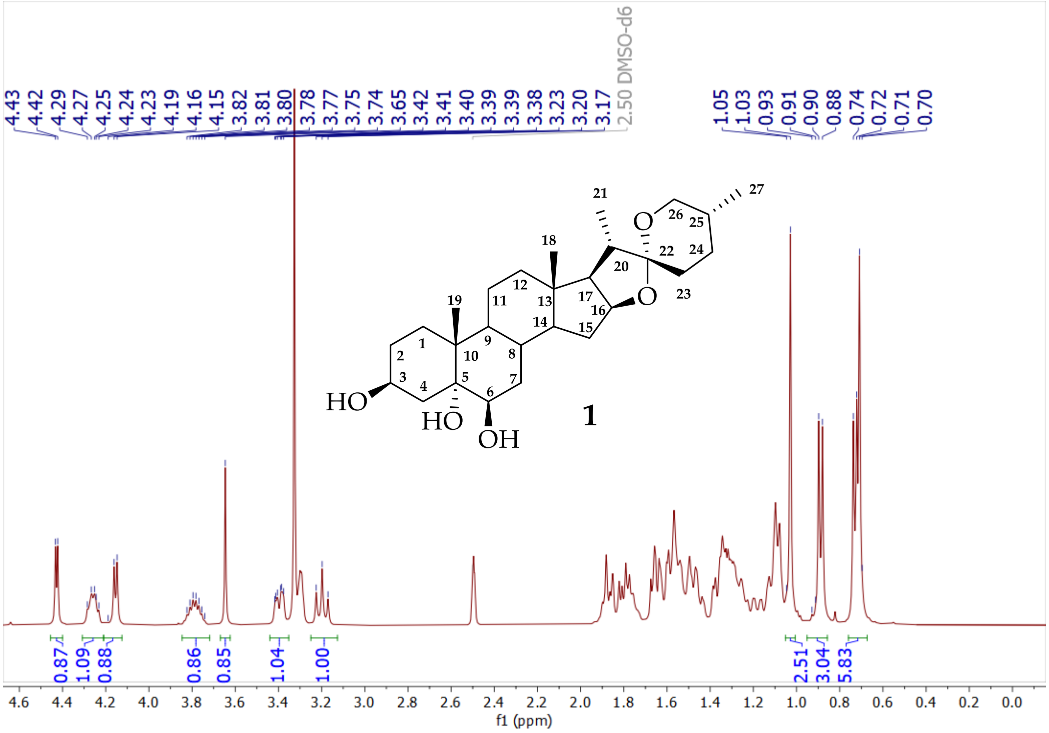

Copyright: © 2023 by the authors. Licensee MDPI, Basel, Switzerland. This article is an open access article distributed under the terms and conditions of the Creative Commons Attribution (CC BY) license (https://creativecommons.org/licenses/by/4.0/).

^{13}C-NMR

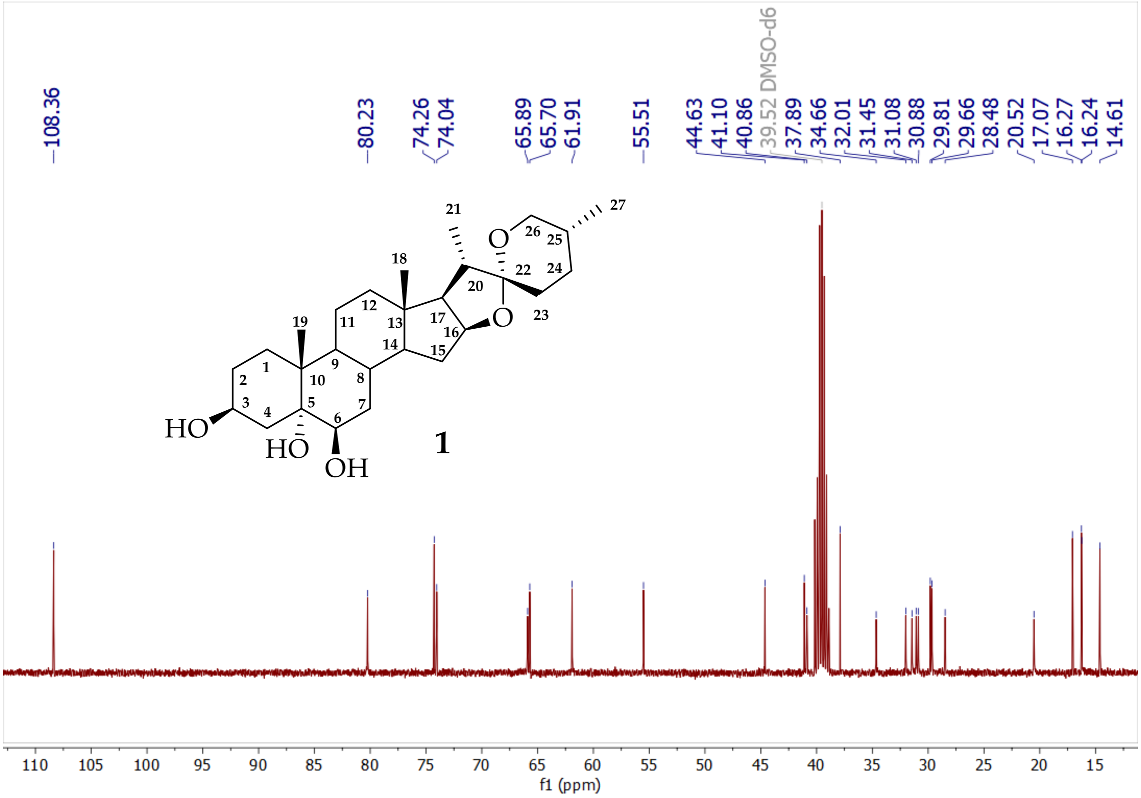

***Figure S2**: NMR spectra of (25R)-3,6-dioxo-5α-spirost-5-ol (**2**)*

^{1}H-NMR

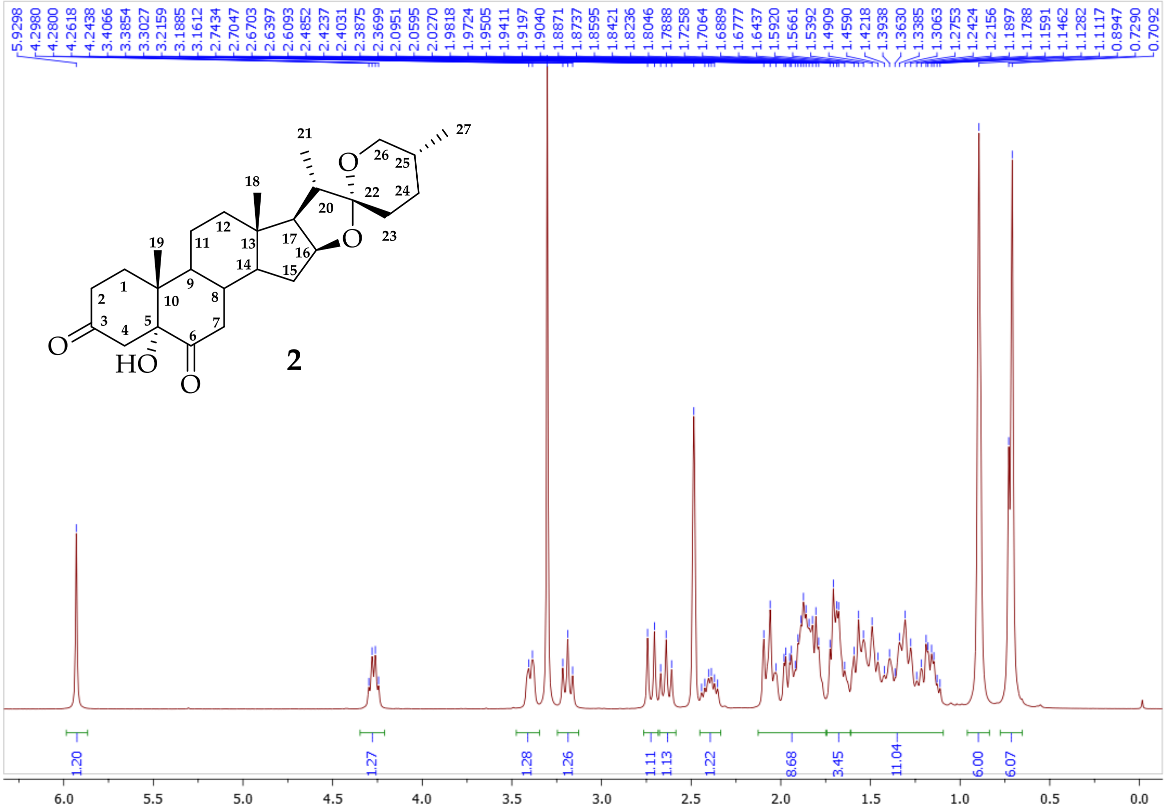

^{13}C-NMR

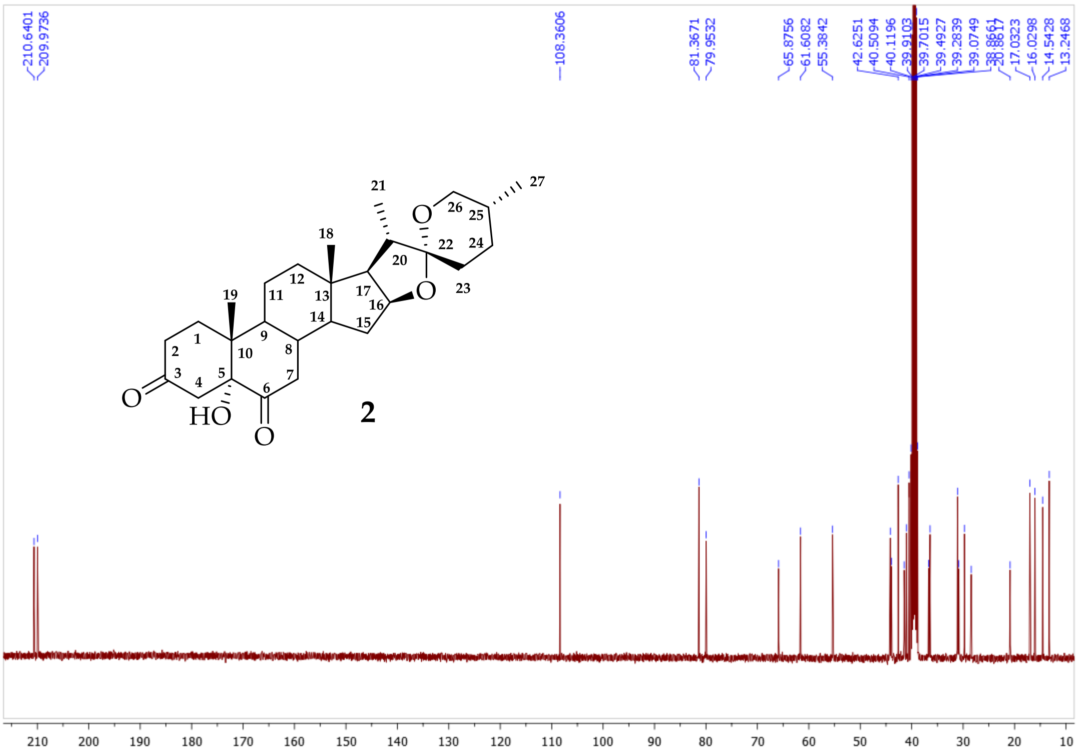

2D COSY

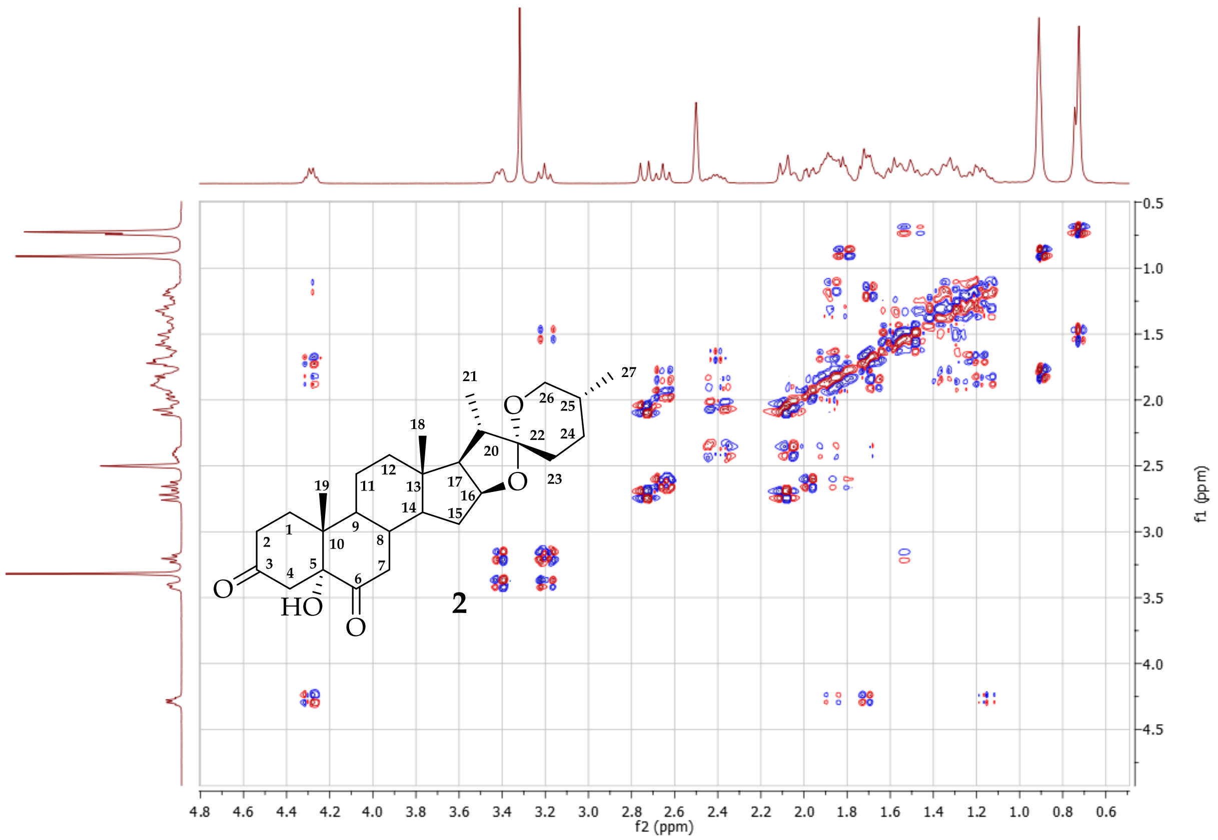

2D NOESY

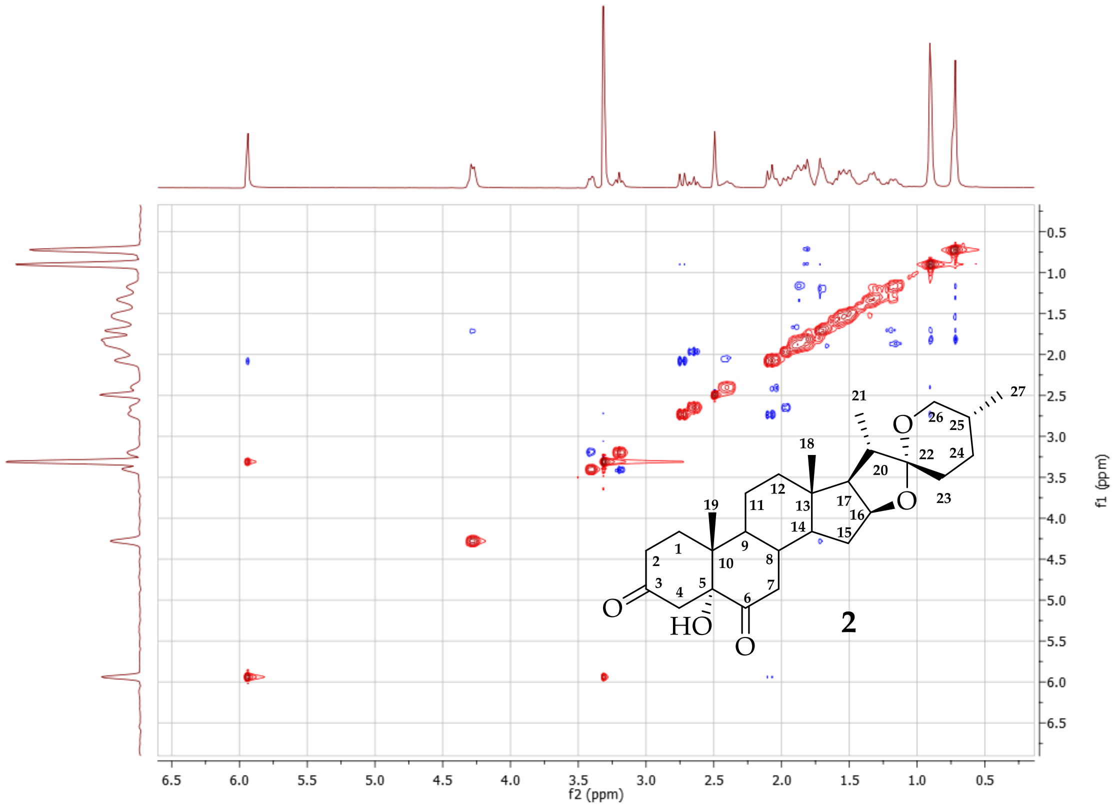

2D HSQC

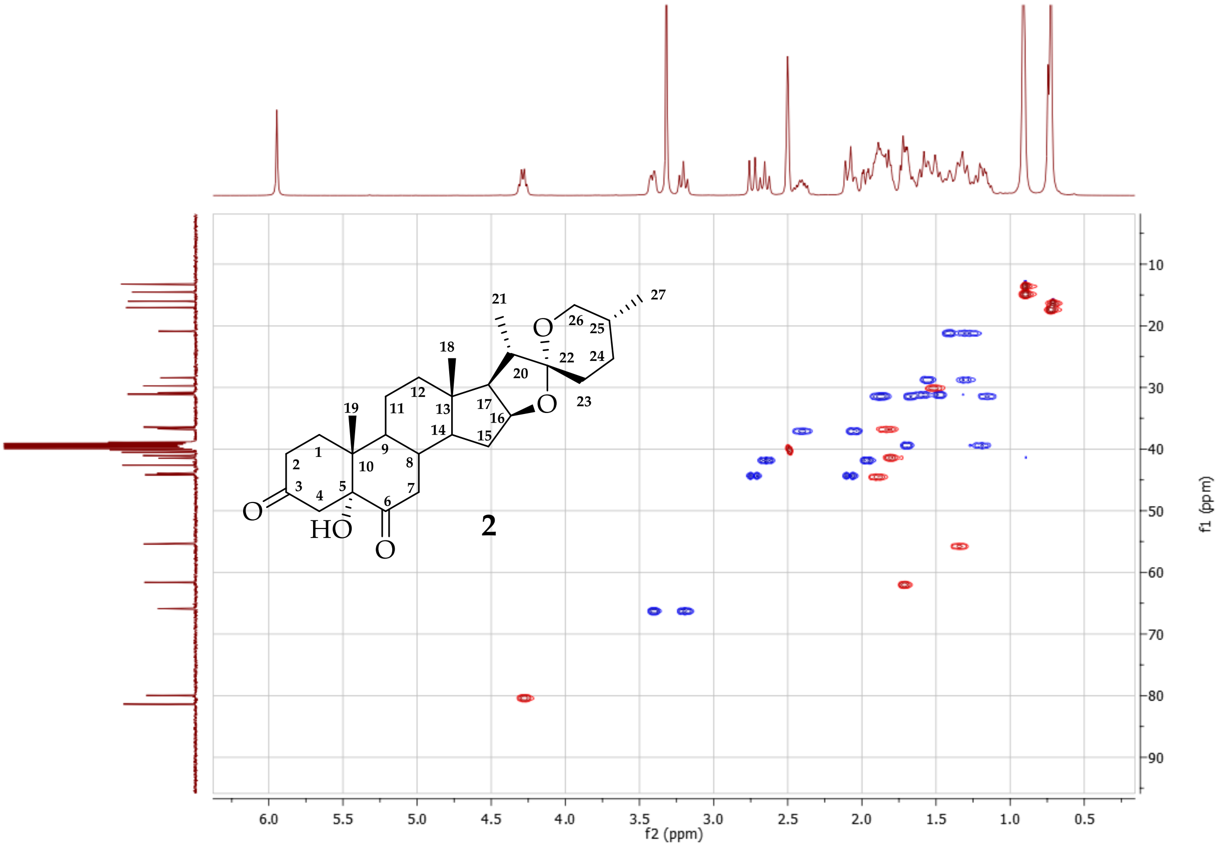

2D HMBC

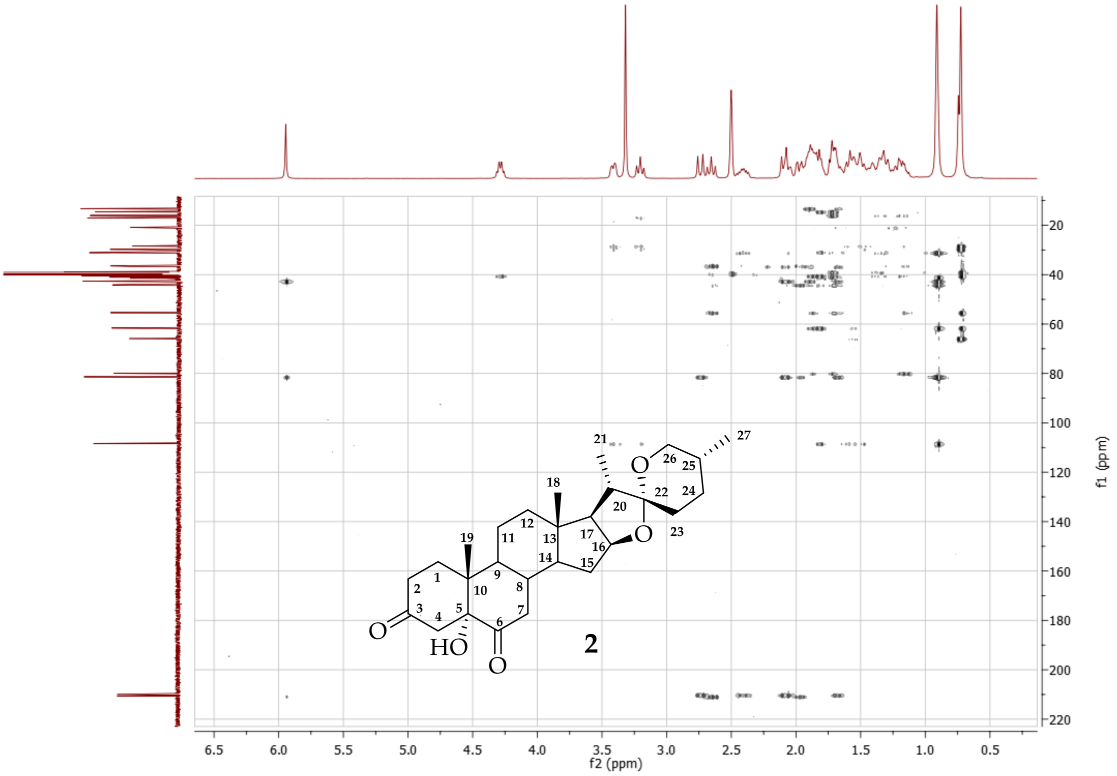

Figure S3: NMR spectra of (25R)-spirost-4 –en-3,6-dione (3)

^{1}H-NMR

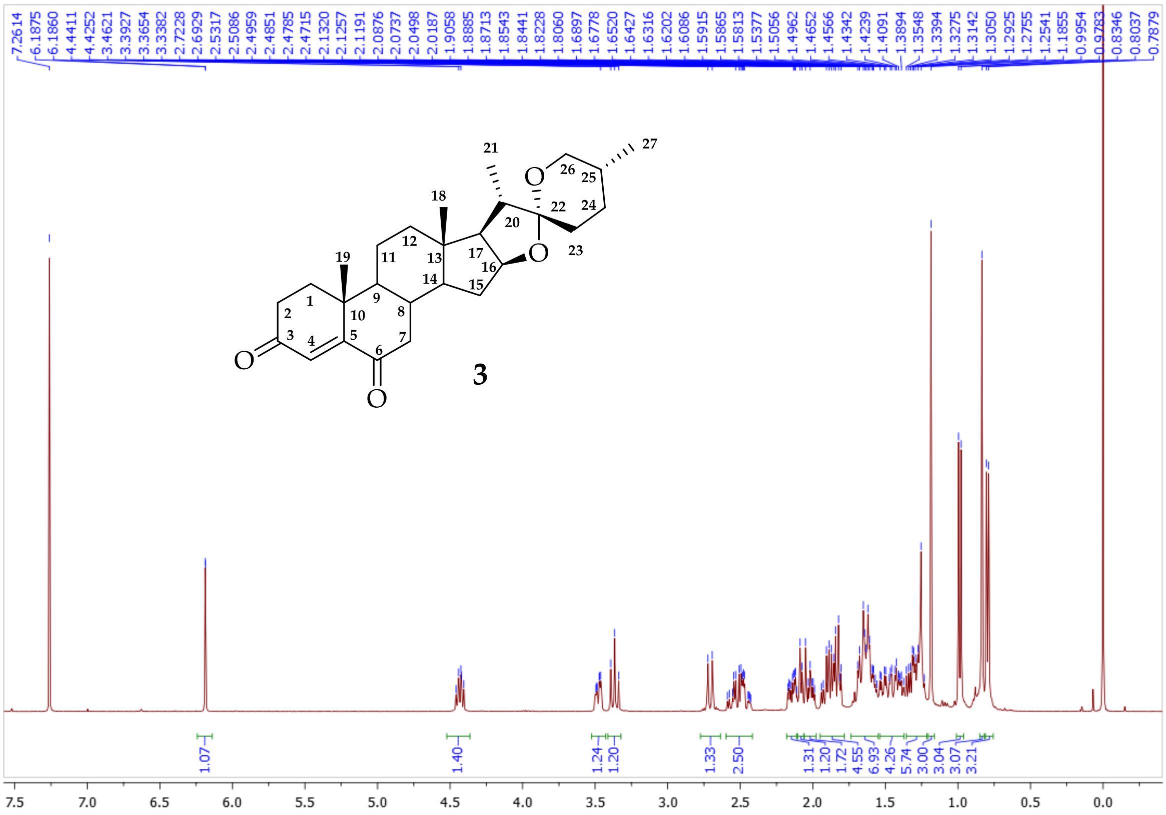

^{13}C-NMR

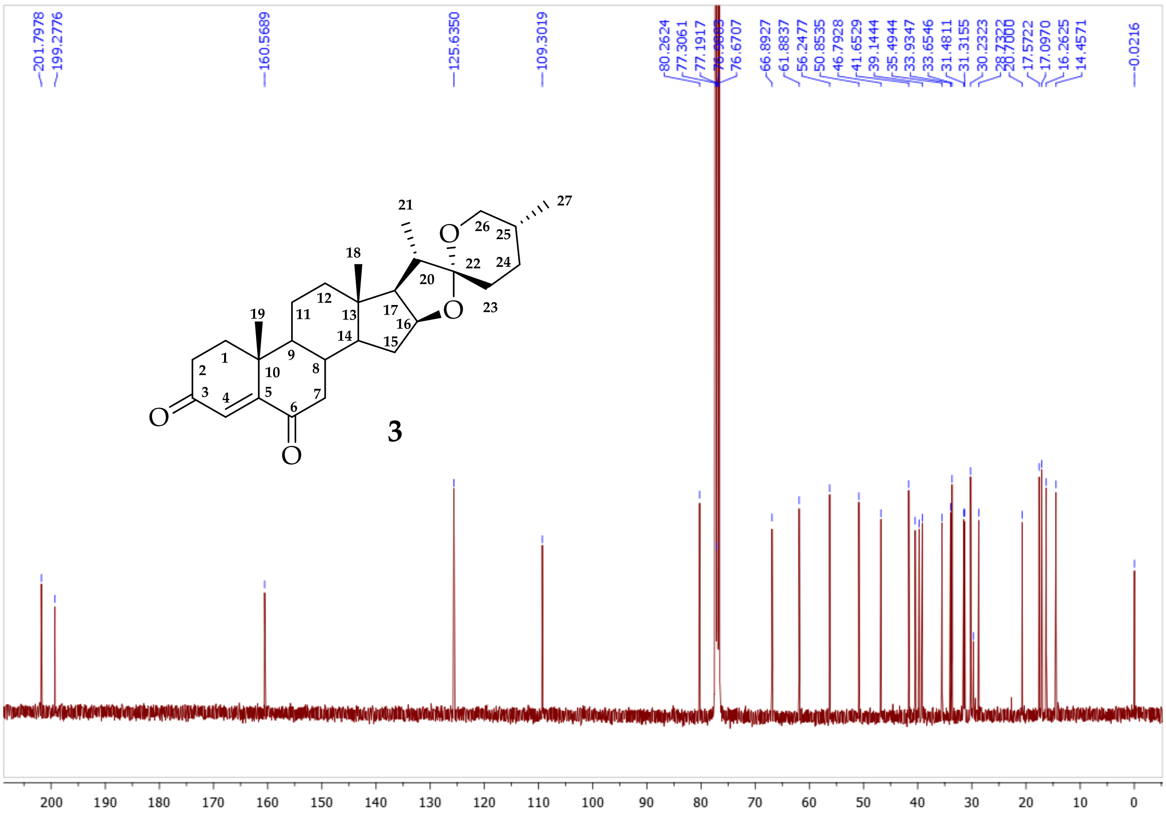

2D COSY

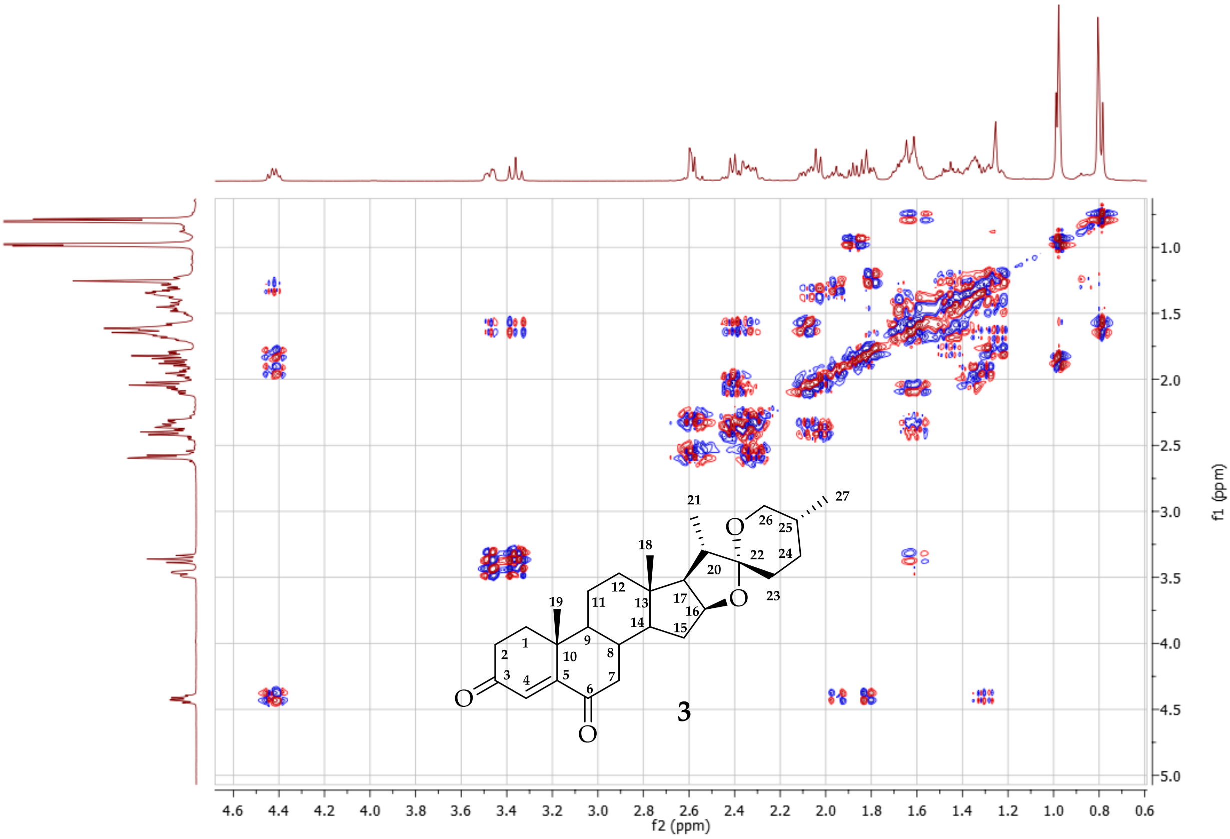

2D NOESY

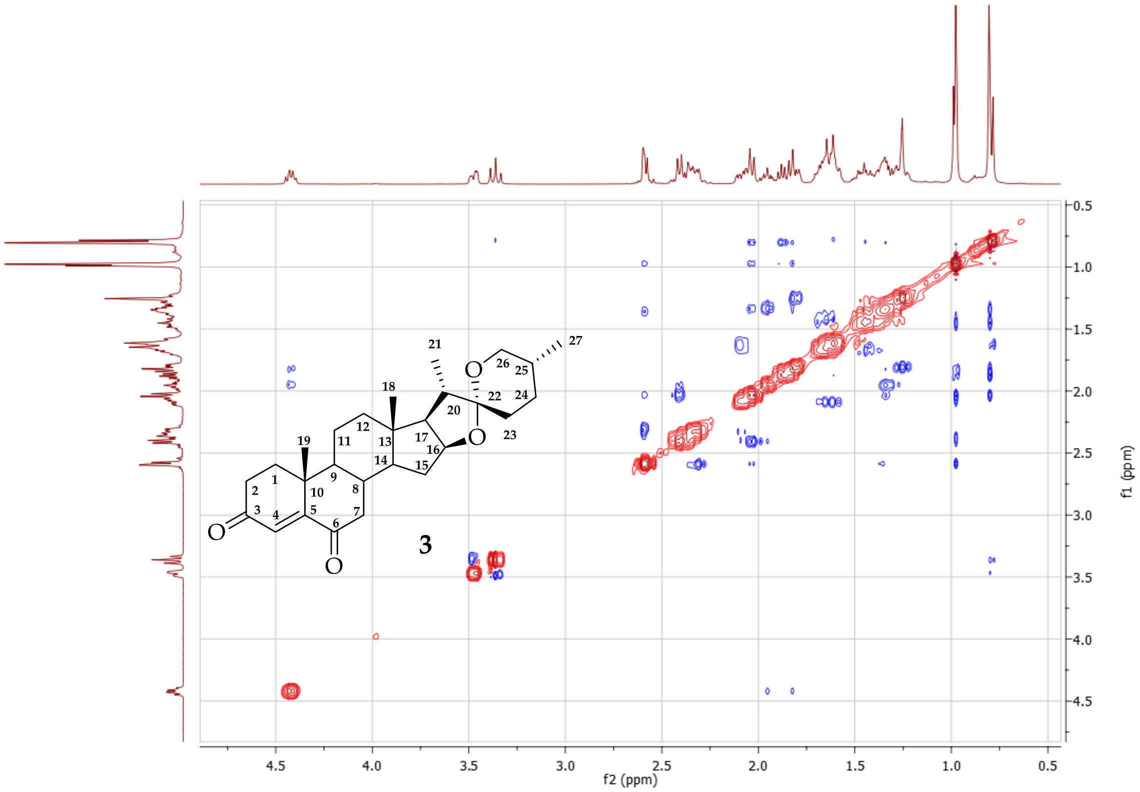

2D TOCSY

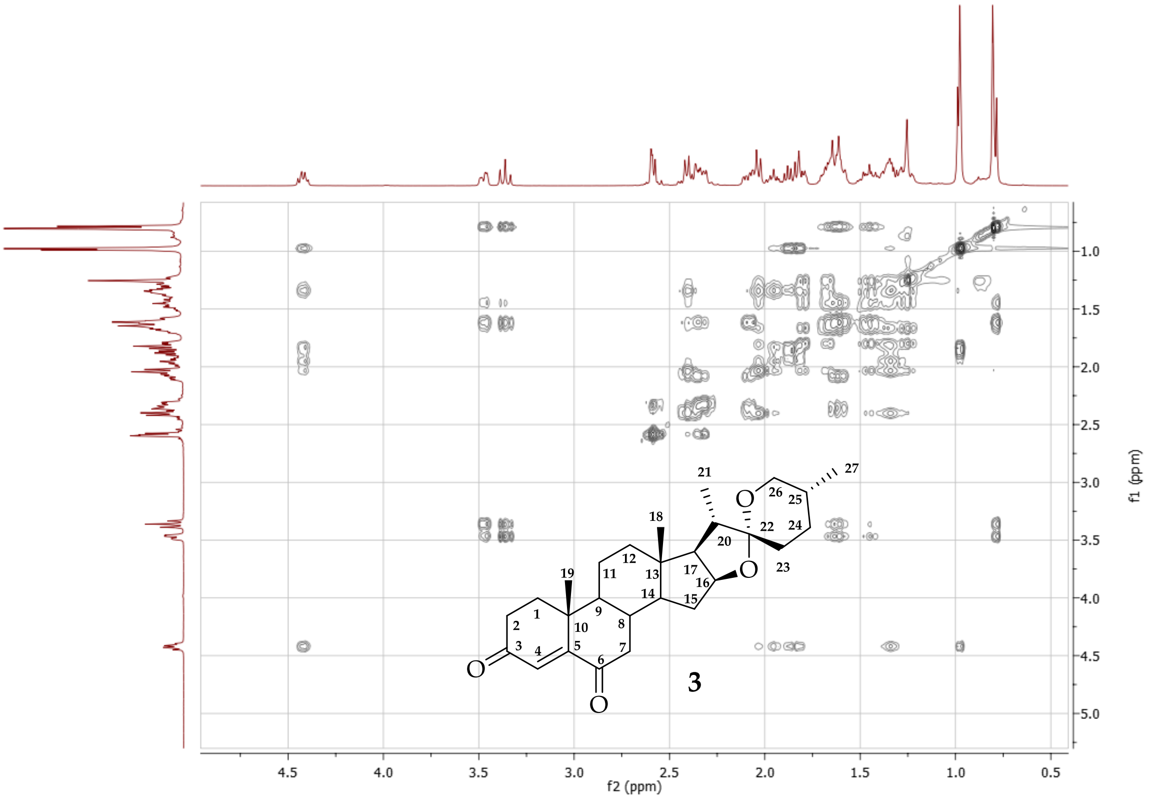

2D HSQC

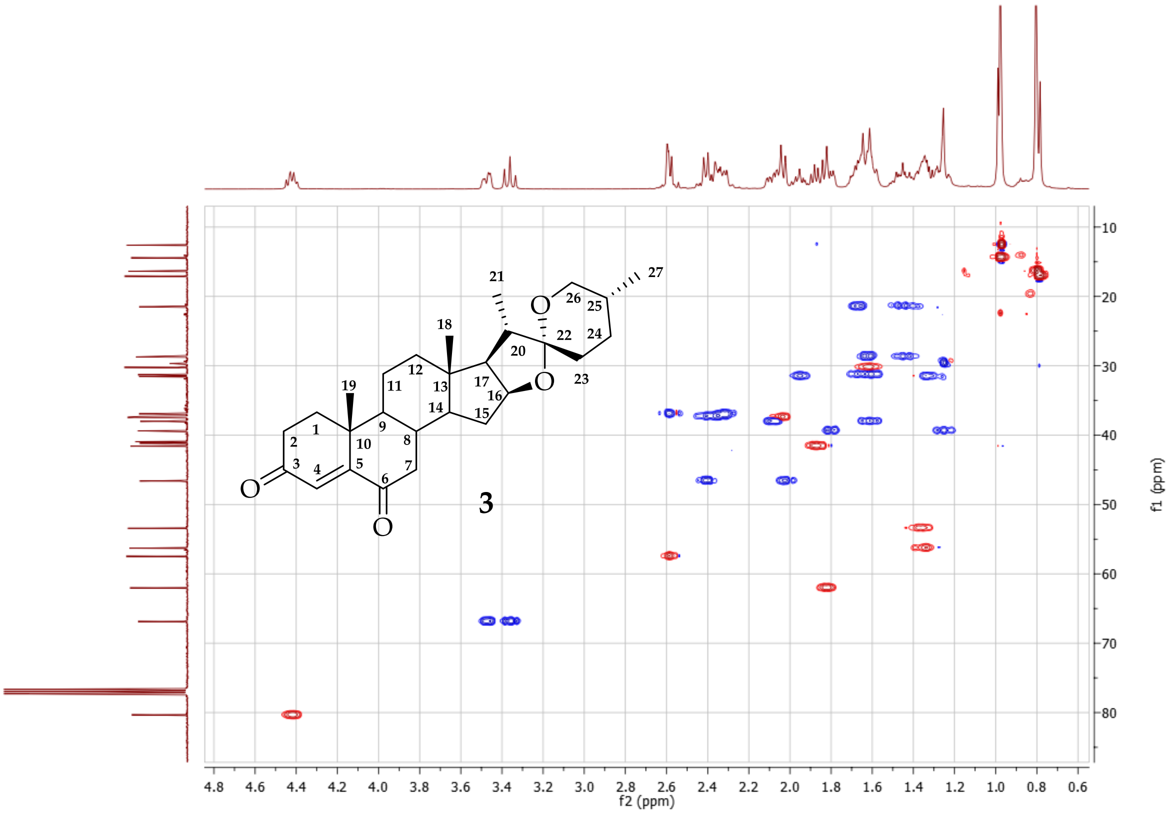

2D HMBC

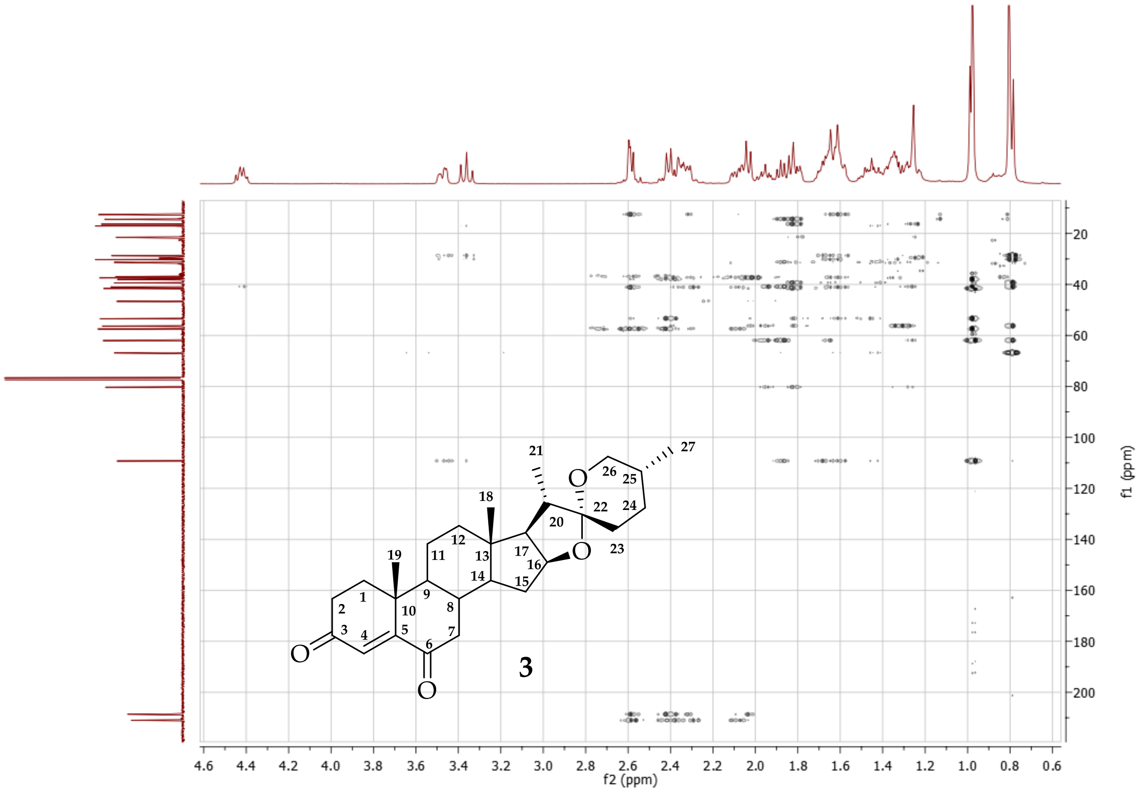

Figure S4: NMR spectra (25R)- 5α-spirost-3,6-dione (4)

^{1}H-NMR

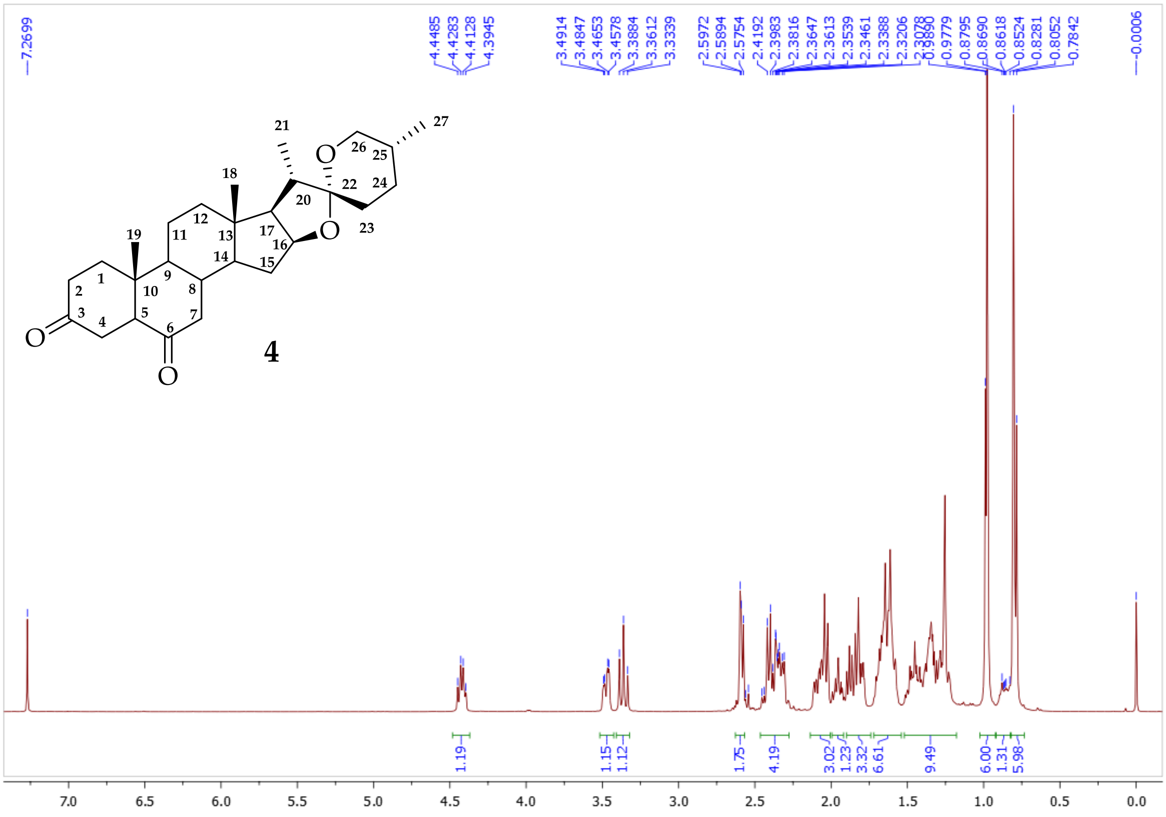

^{13}C-NMR

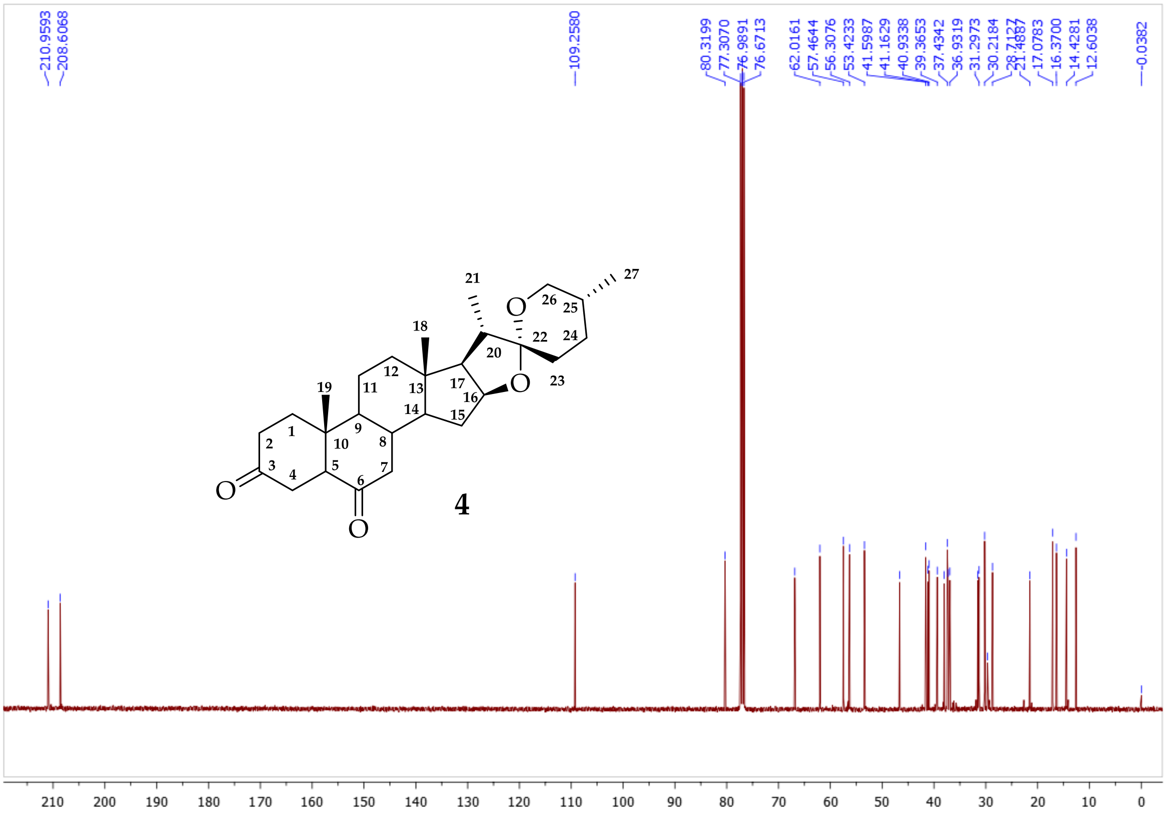

2D COSY

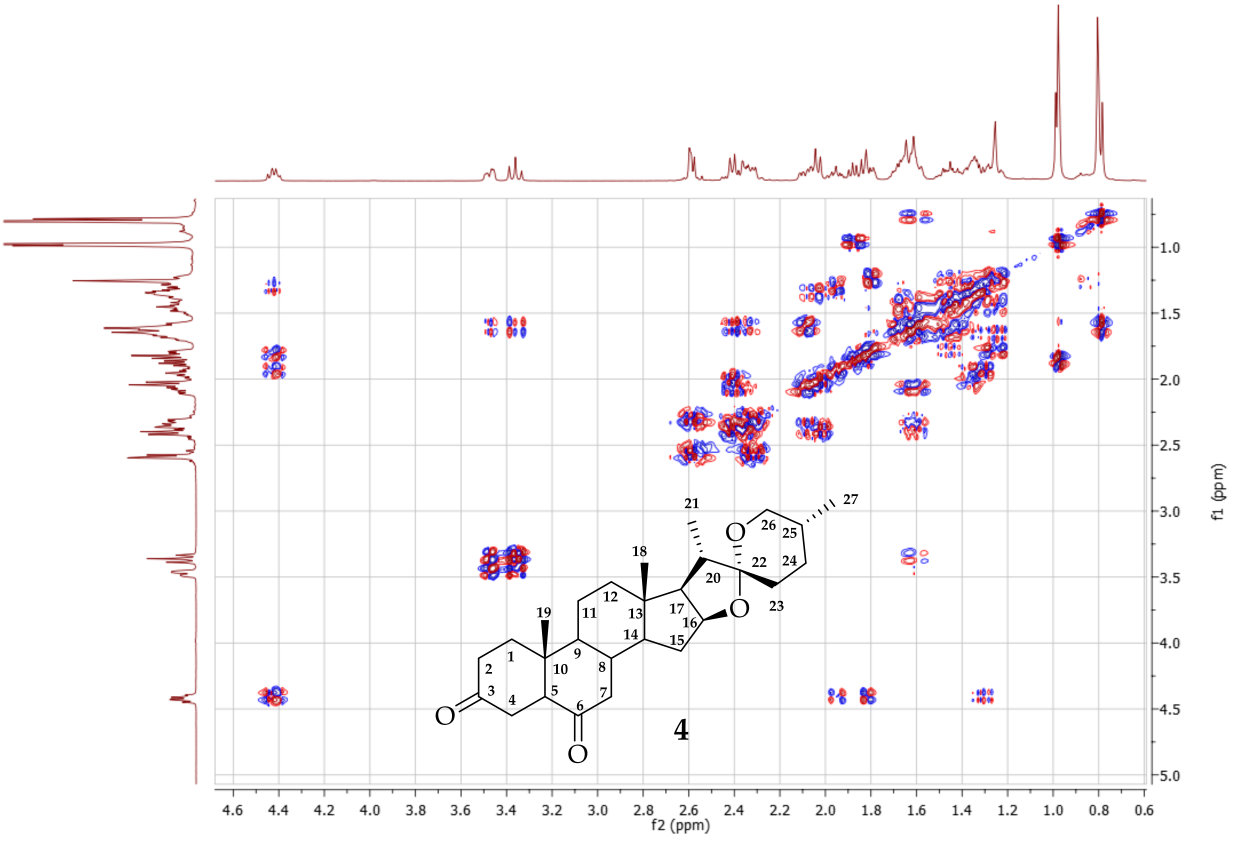

2D NOESY

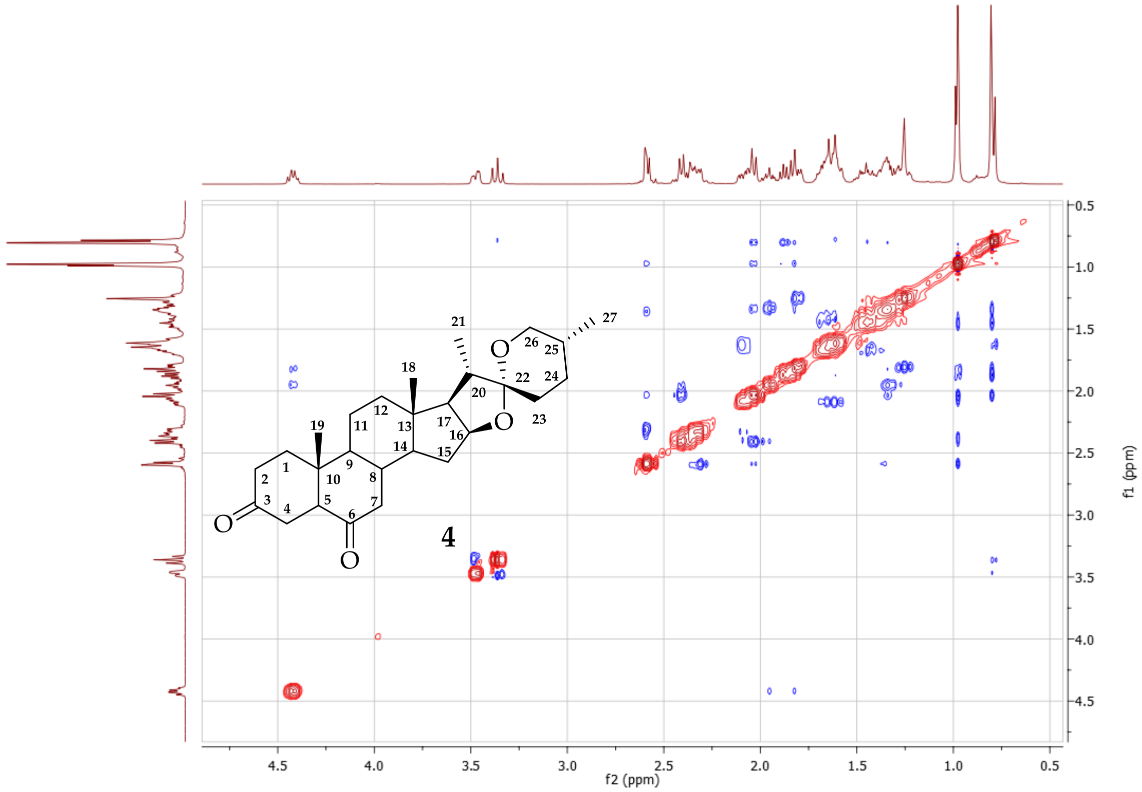

2D TOCSY

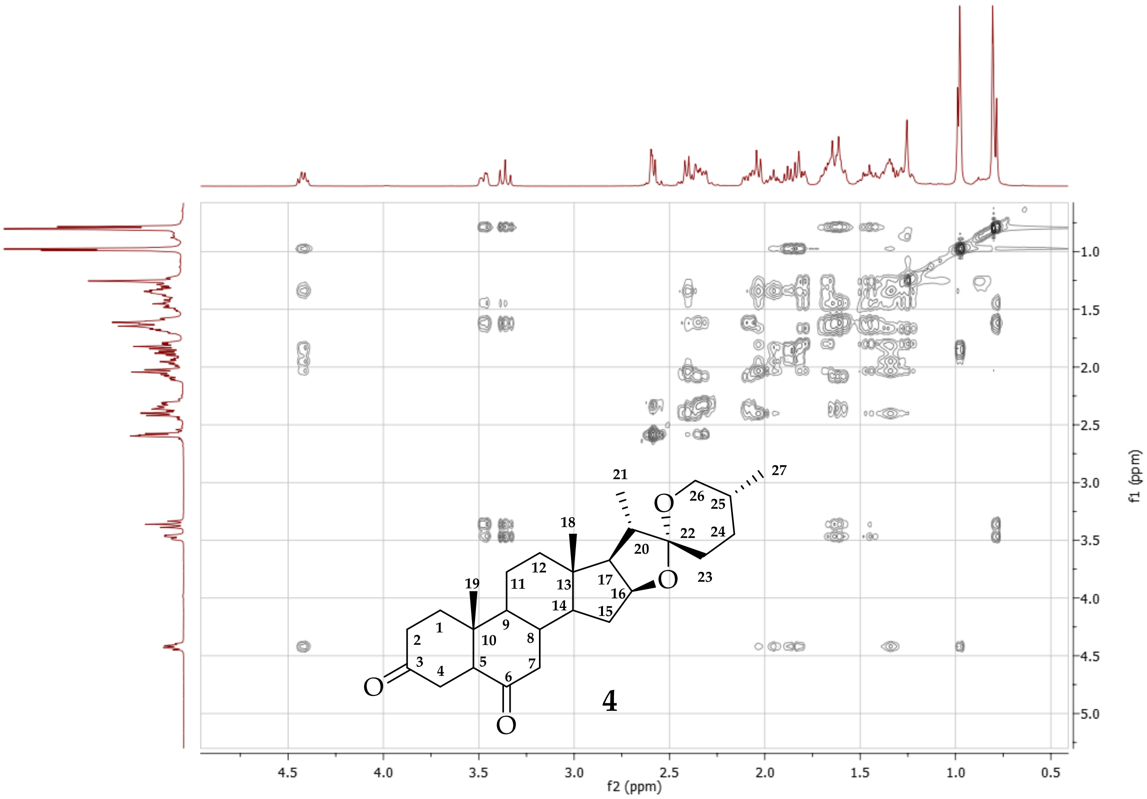

2D HSQC

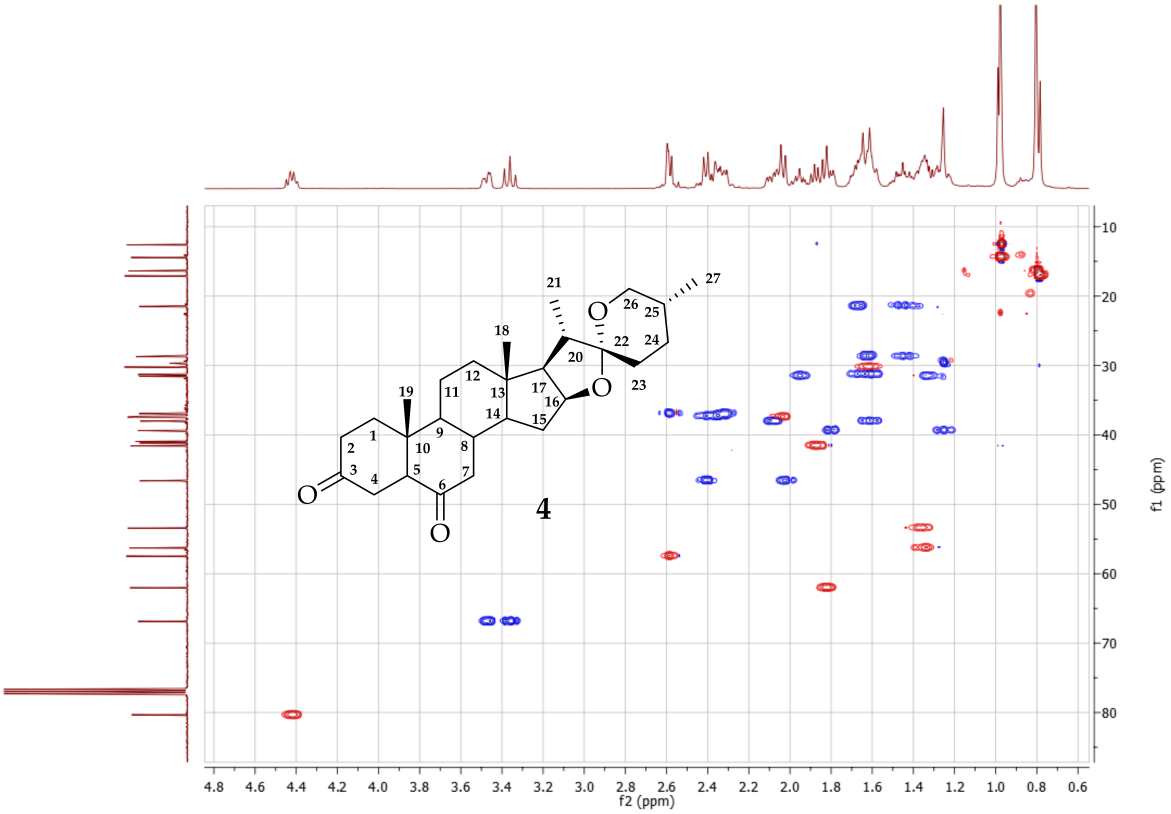

2D HMBC

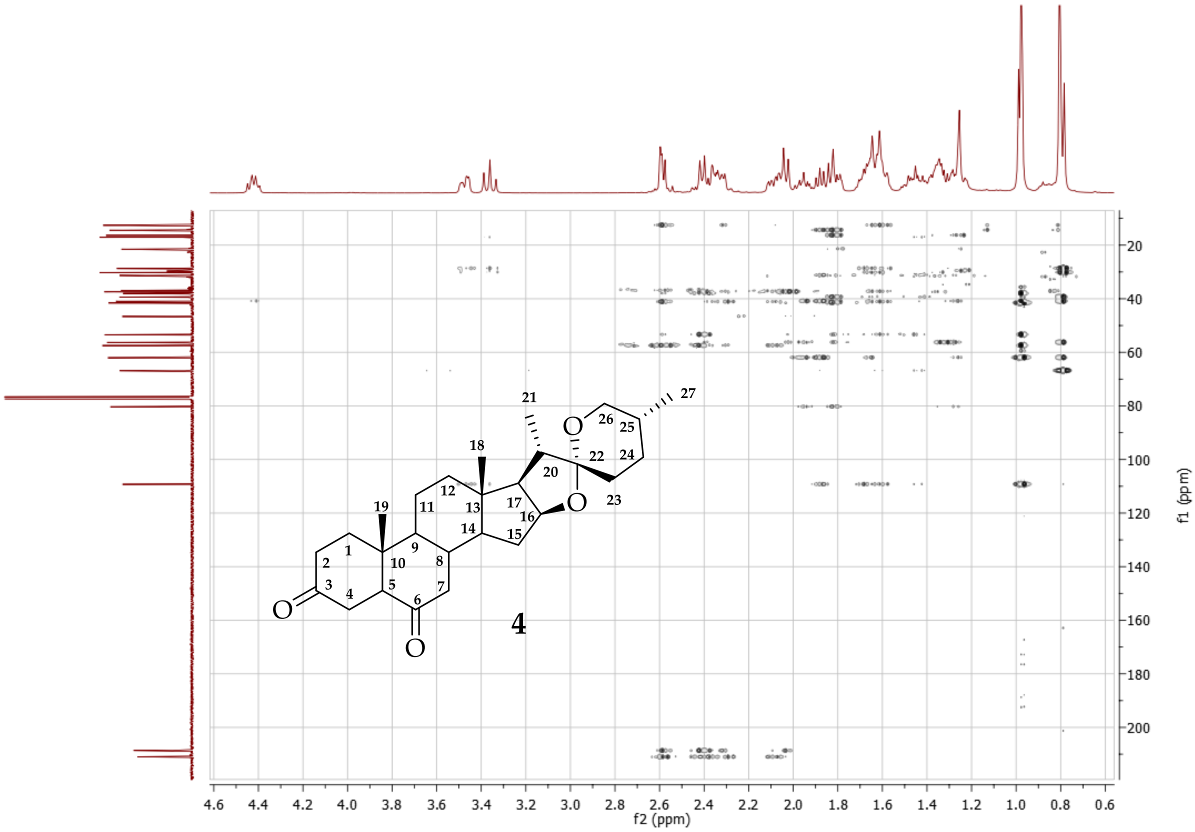

Figure S5*: NMR spectra of (25R)- (3E/Z)-hydroximino-5α-spirost-5-hydroxy-6-ona (****5****)*

^{1}H-NMR

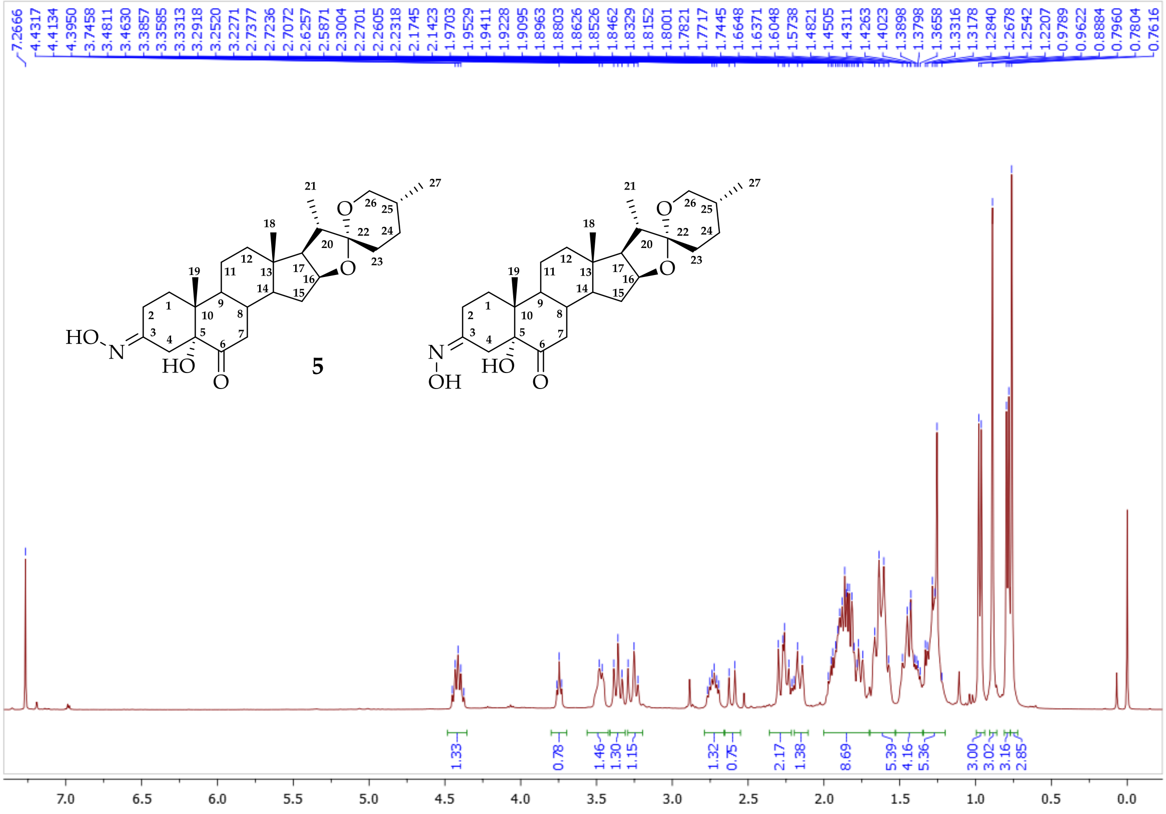

^{13}C-NMR

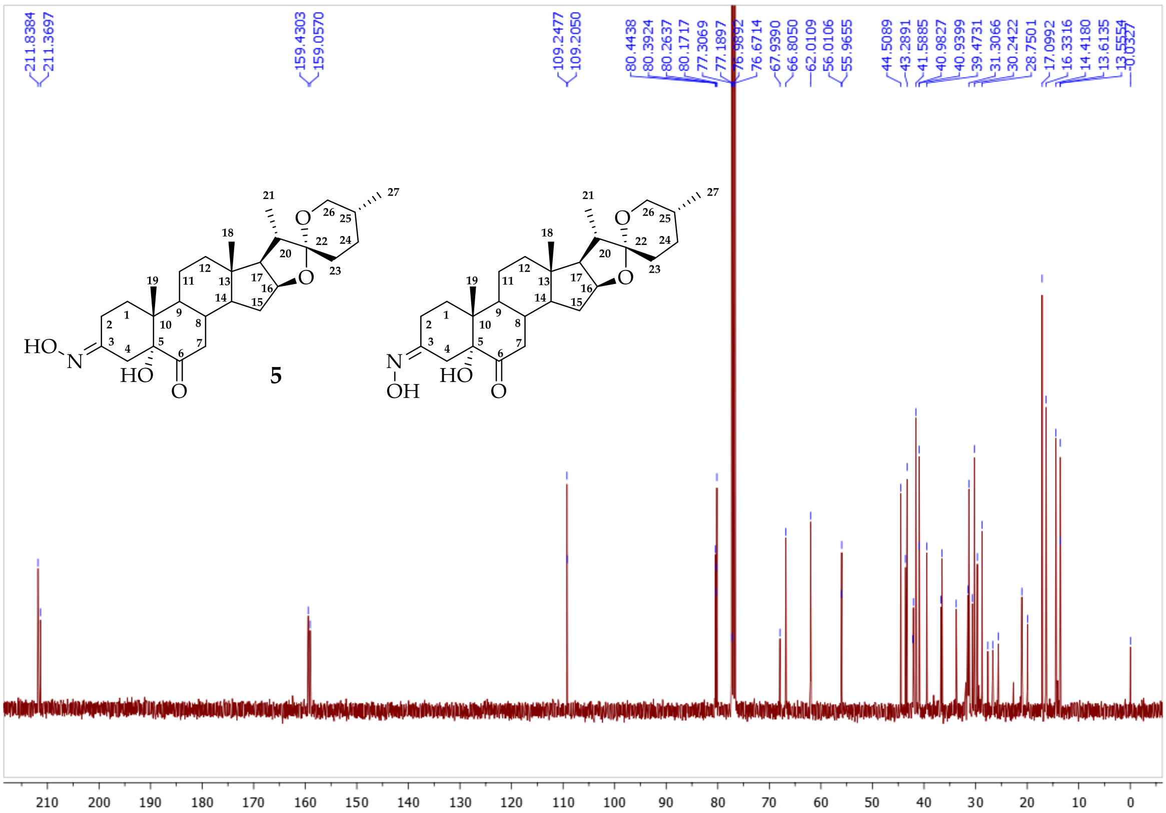

2D COSY

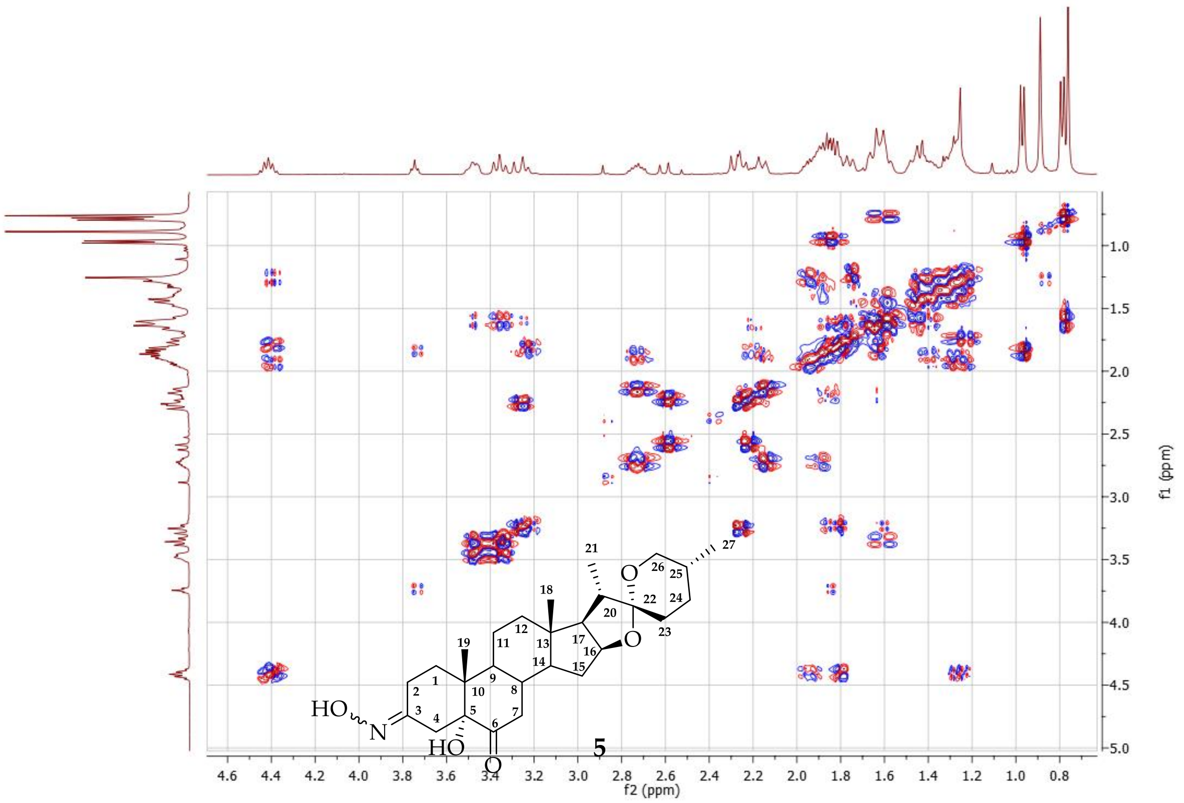

2D TOCSY

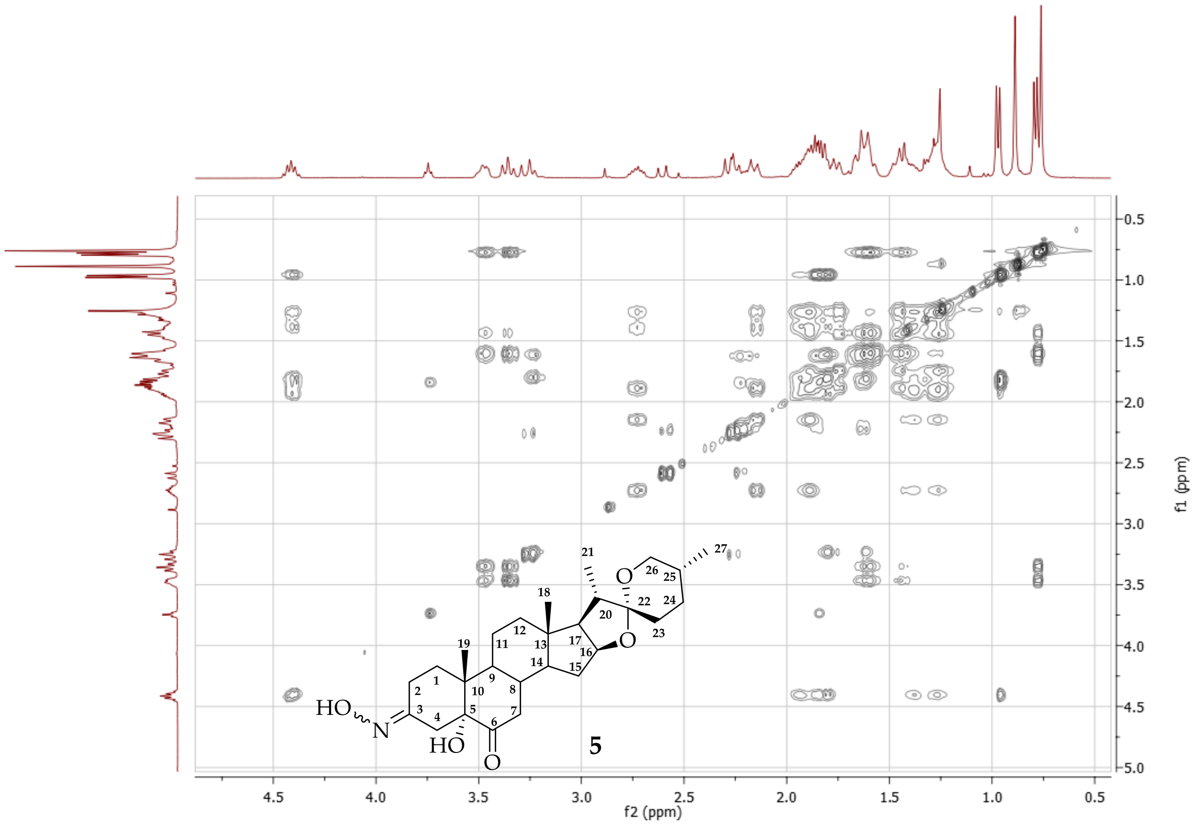

2D NOESY

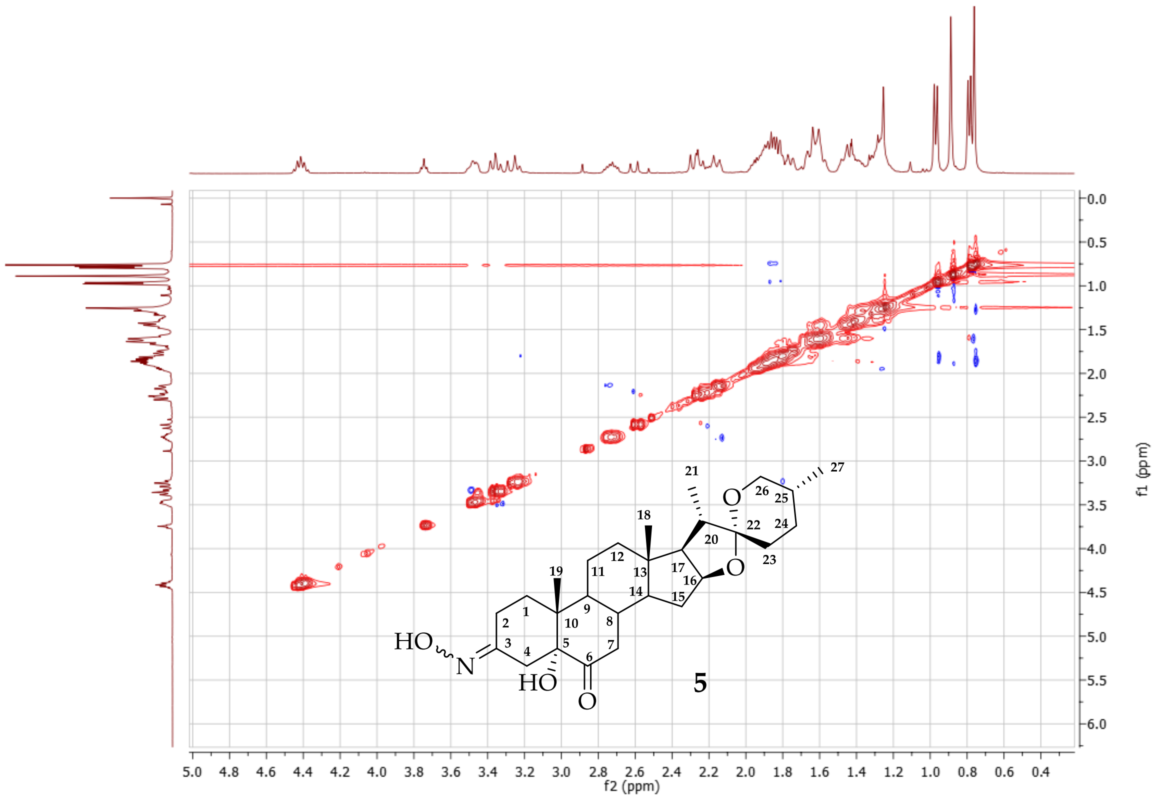

2D HSQC

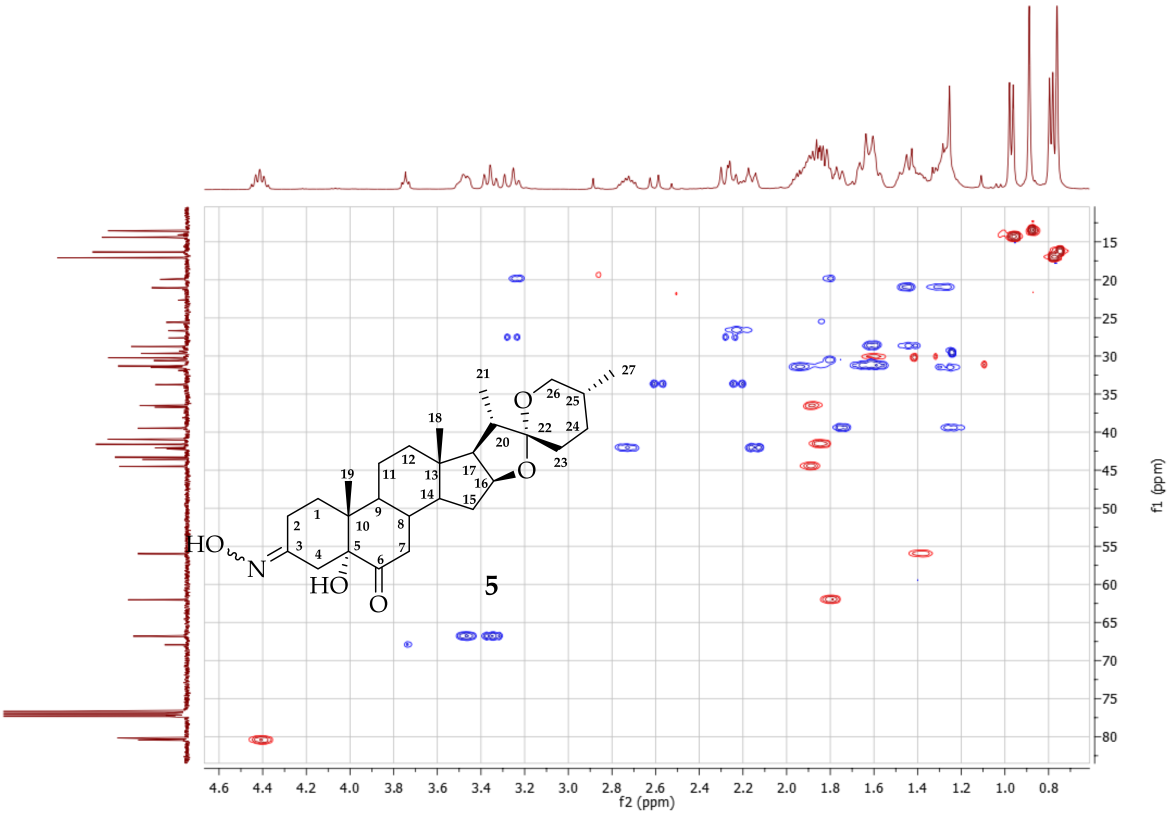

2D HMBC

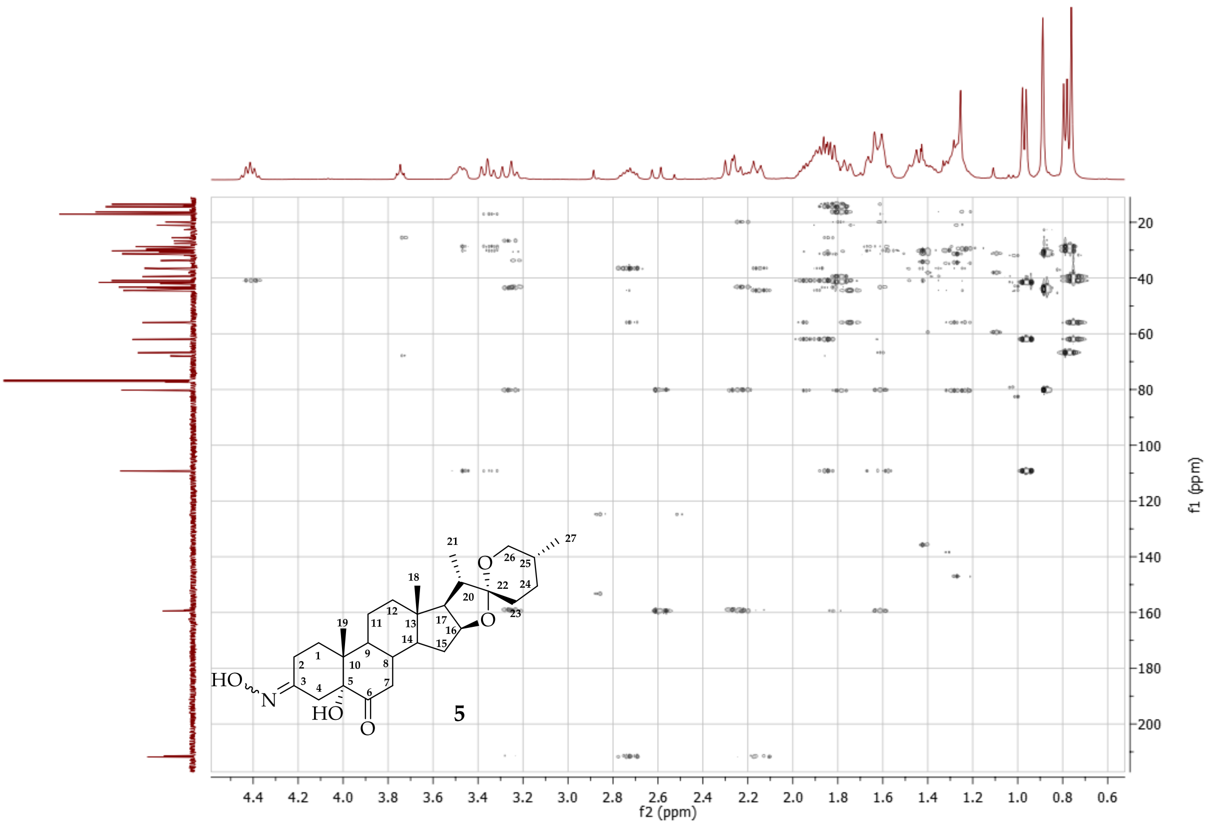

Figure S6: *NMR spectra of (25R)- (3E,6E)-dihydroximinospirost-4-ene (**6**)*

^{1}H-NMR

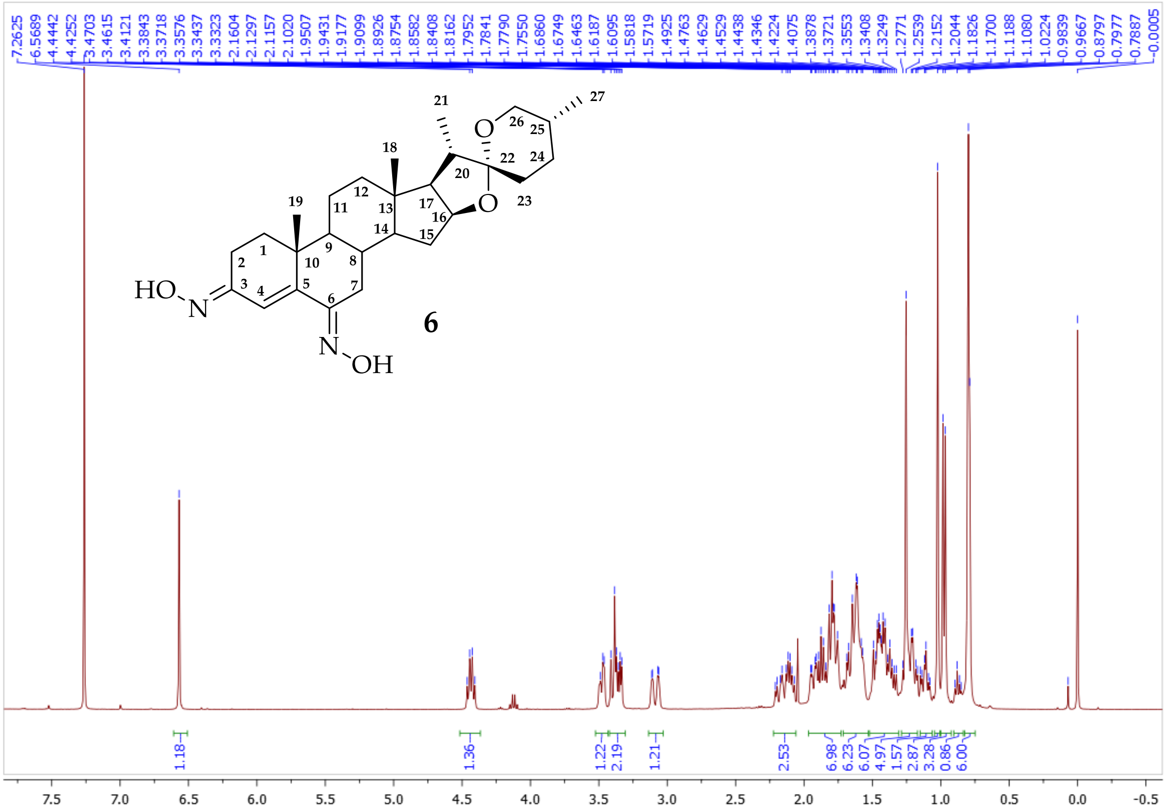

^{13}C-NMR

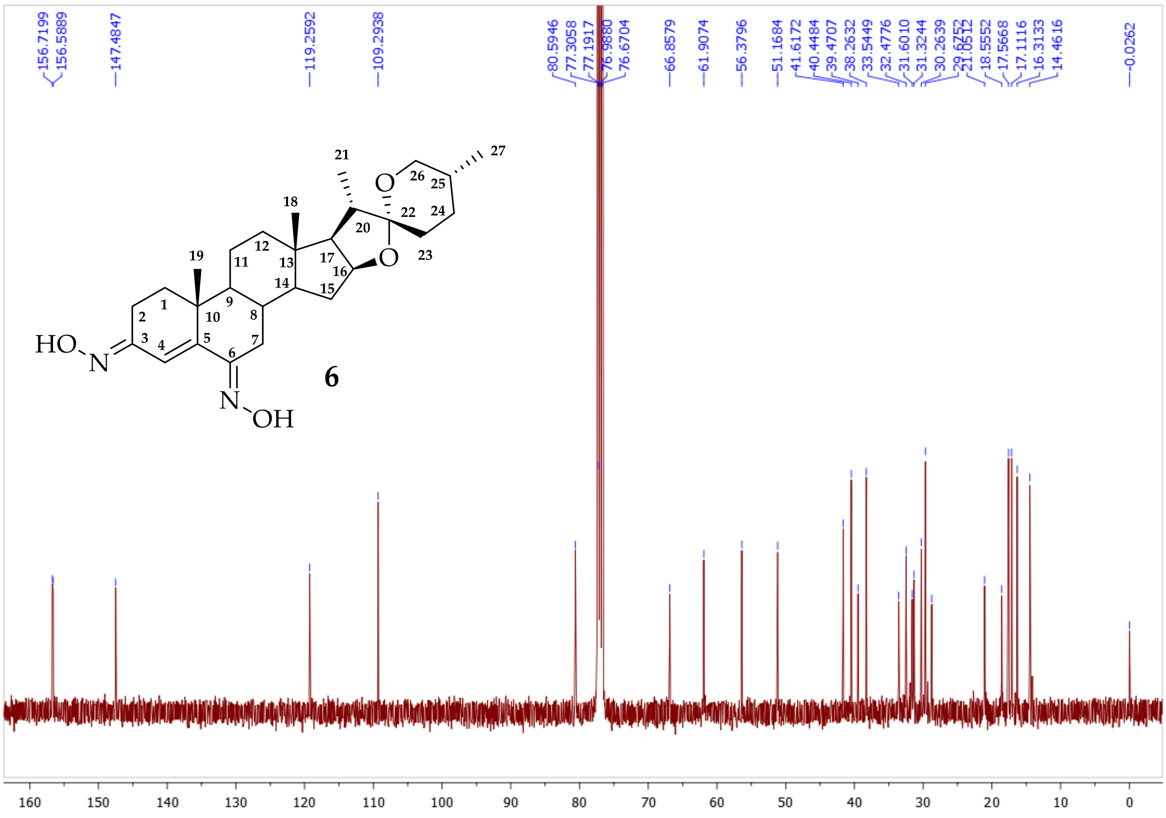

2D COSY

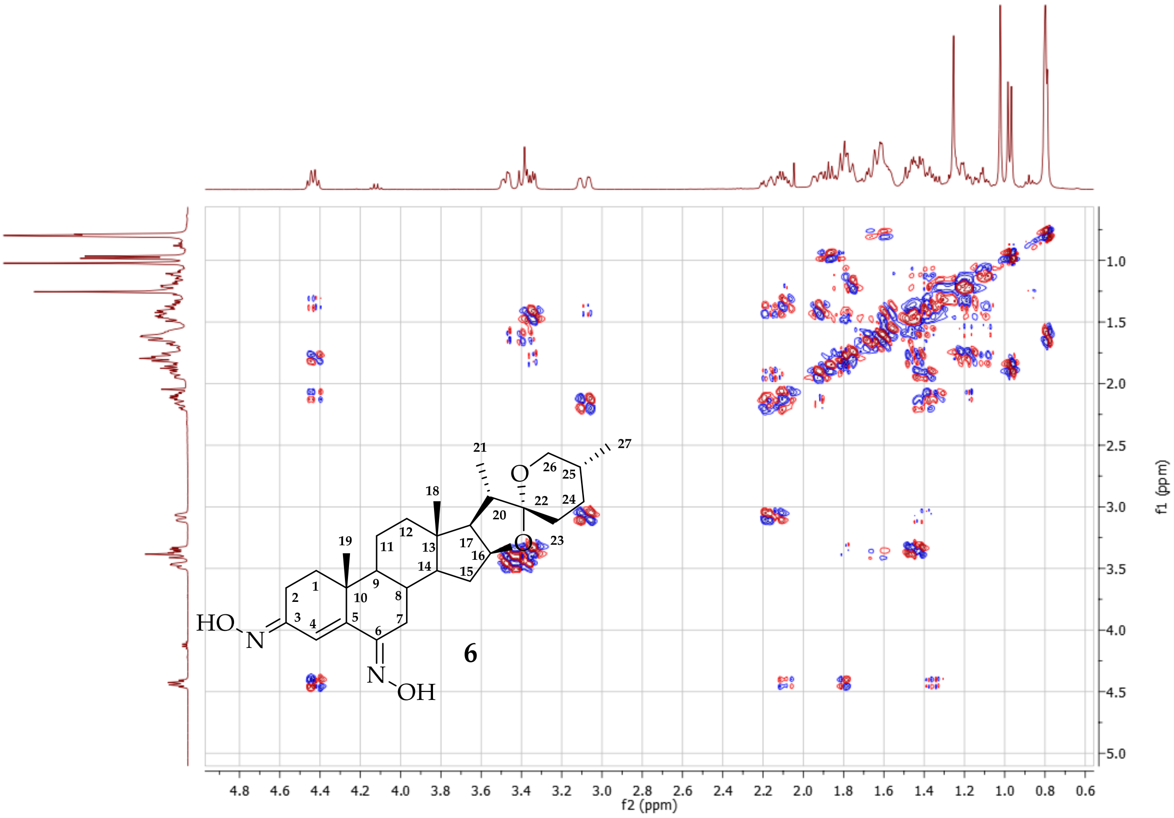

2D ROESY

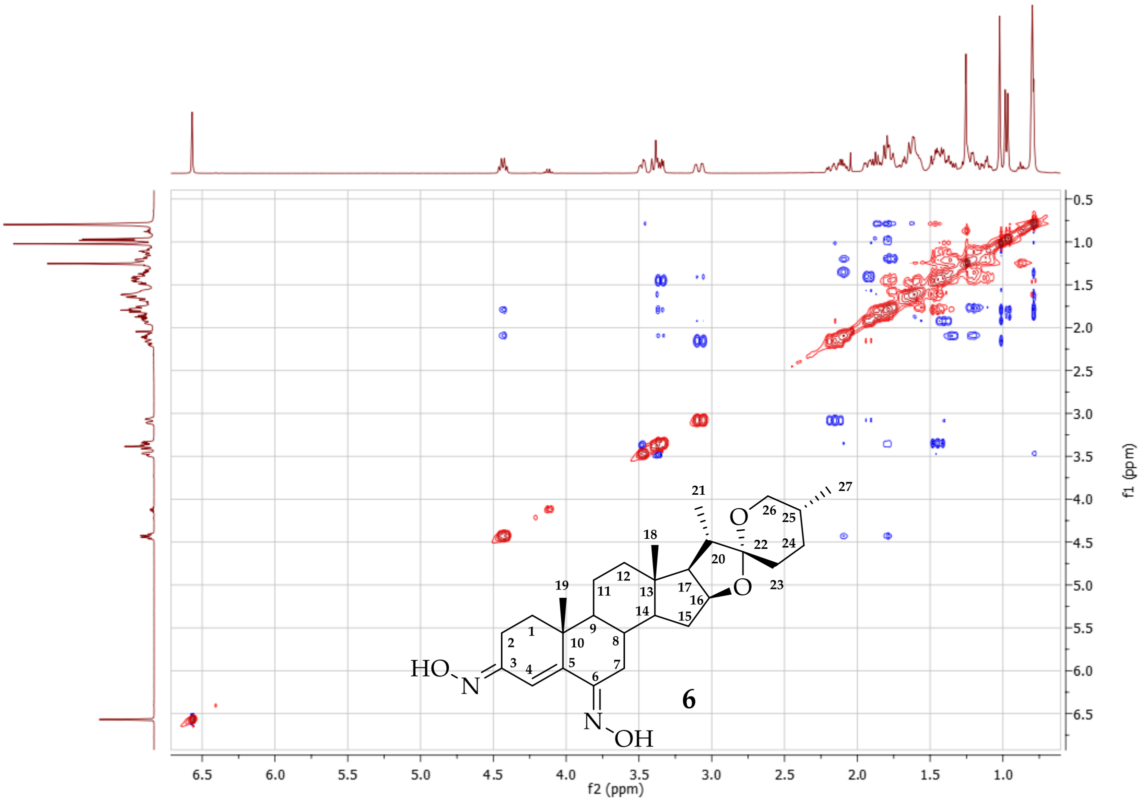

2D HSQC

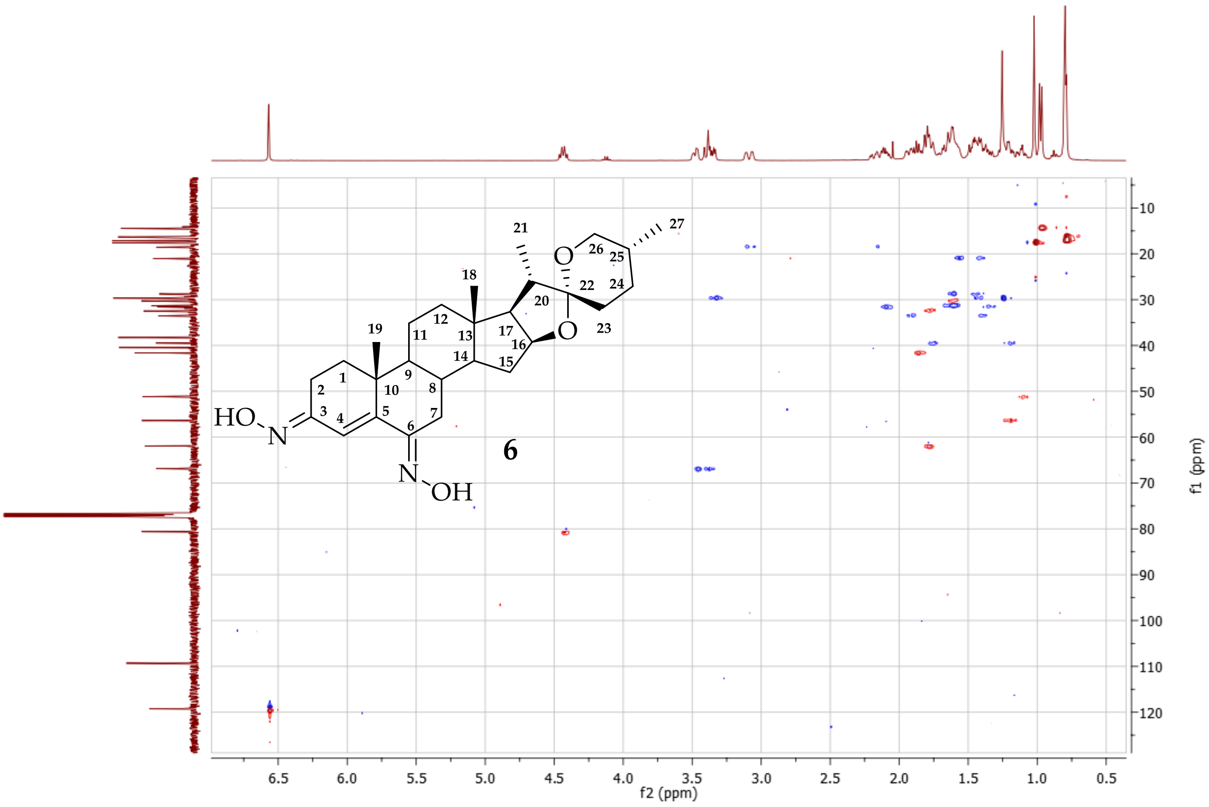

2D HMBC

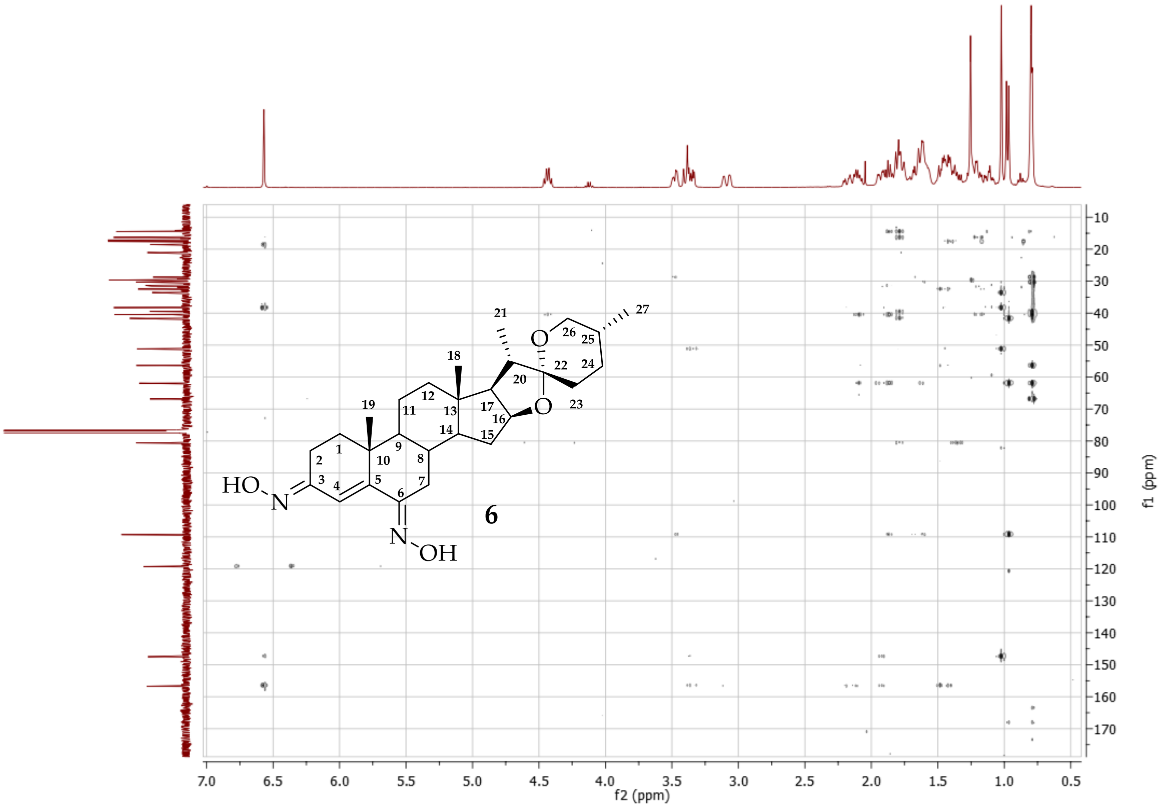

Figure S7*: NMR spectra of mixture (25R)- (3E/Z)-hydroximino-5α-spirost-6-ona (****7****)*

^{1}H-NMR

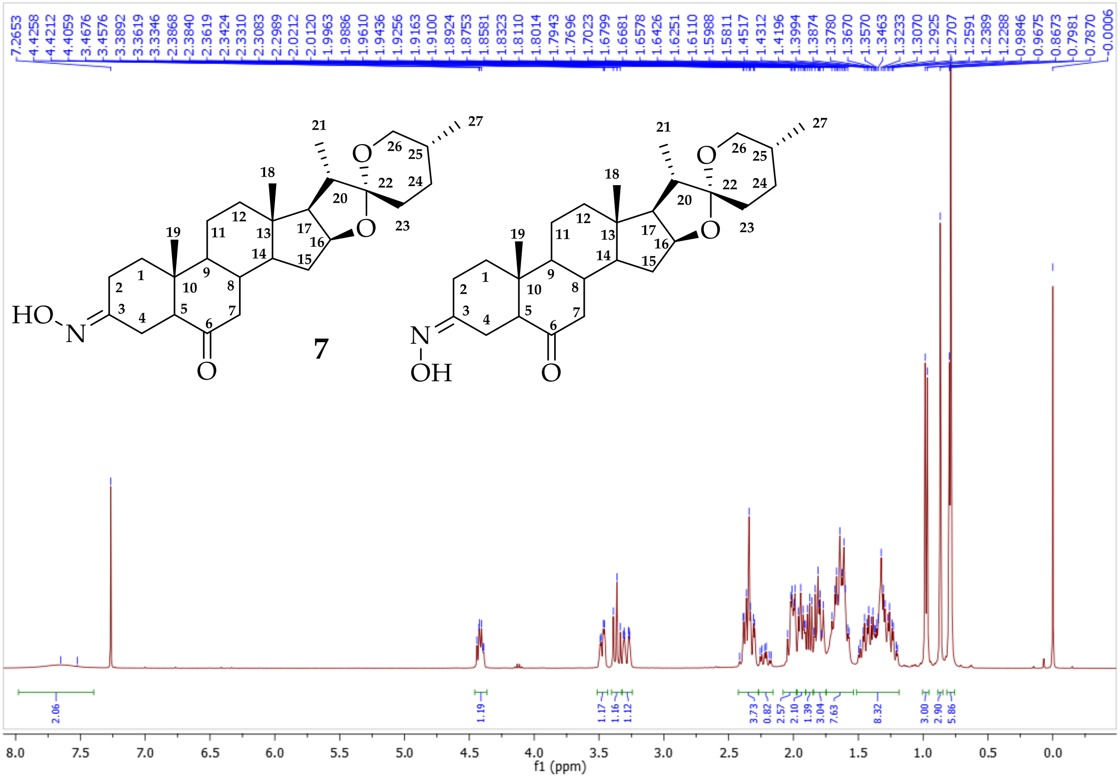

^{13}C-NMR

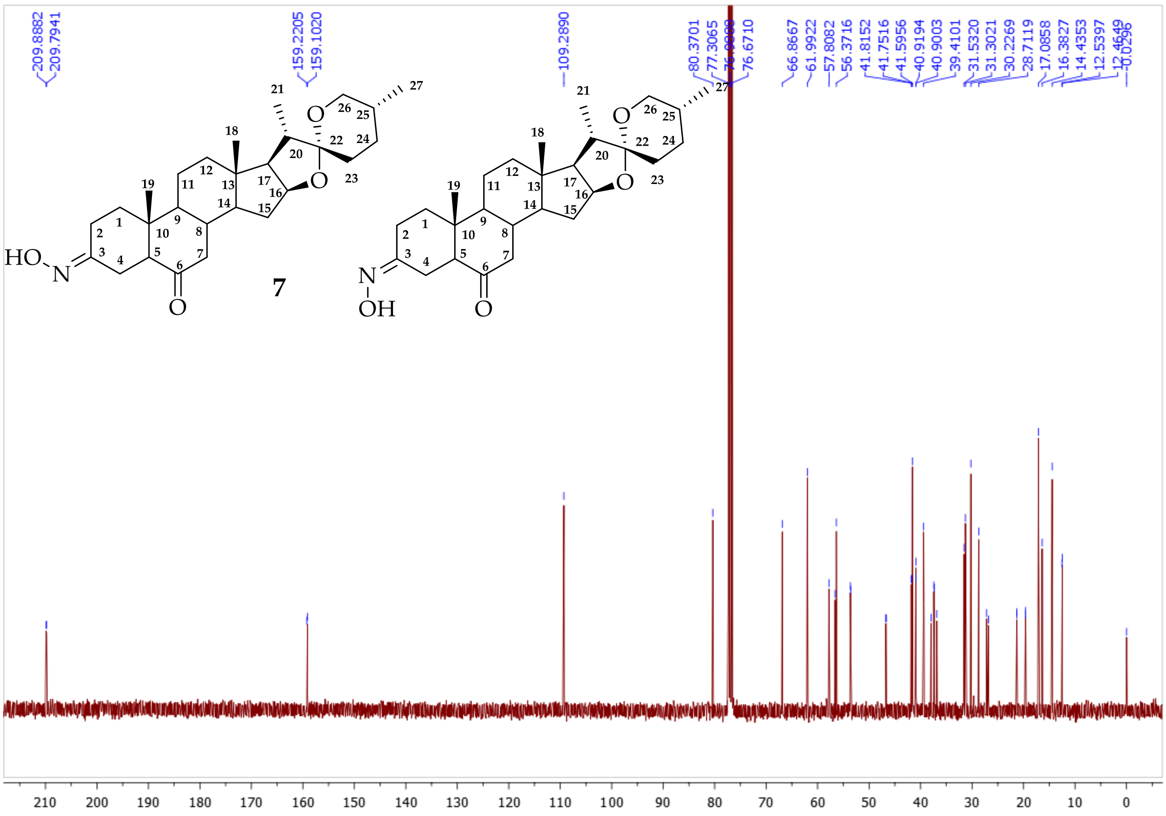

2D COSY

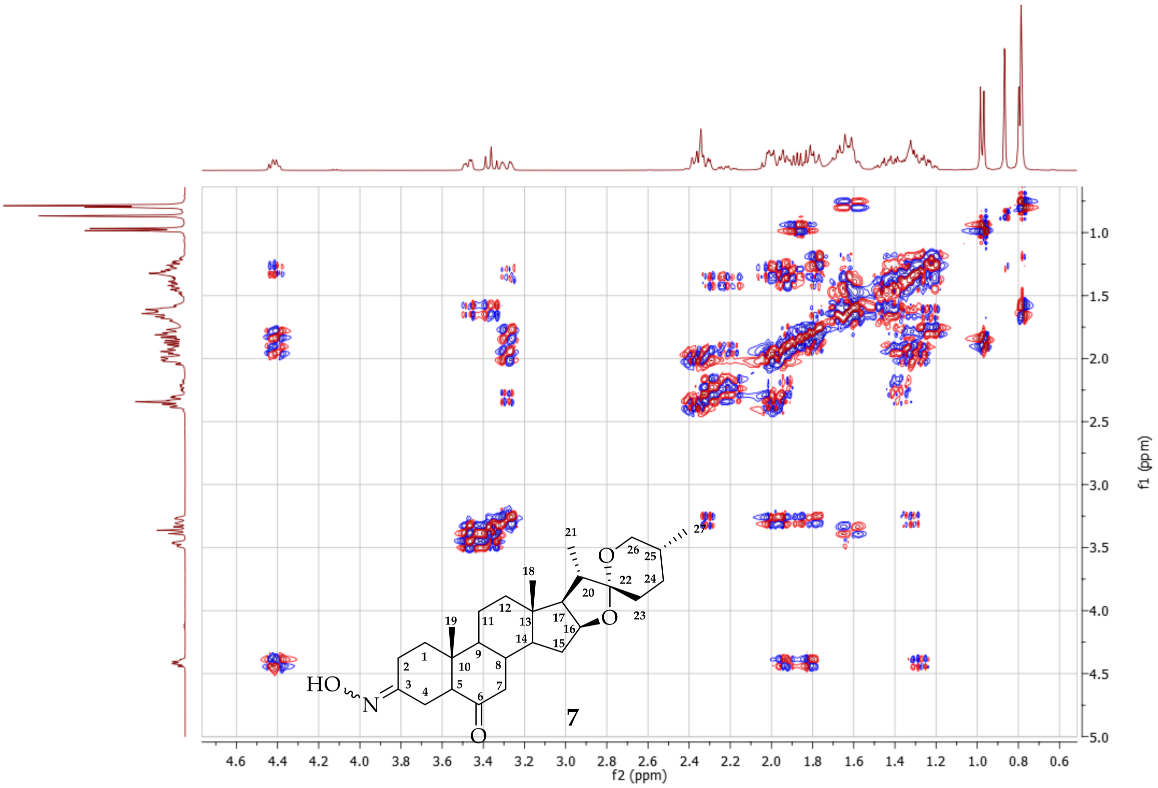

2D NOESY

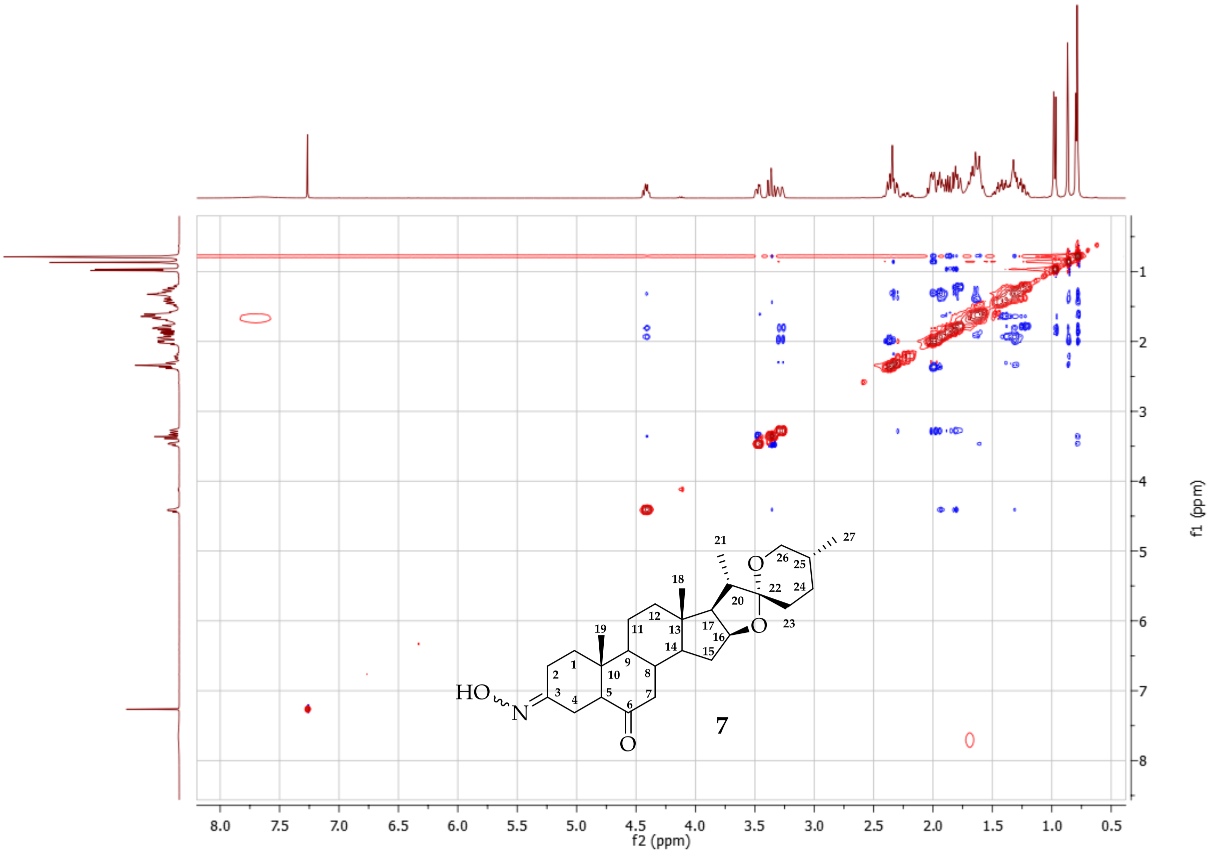

2D HSQC

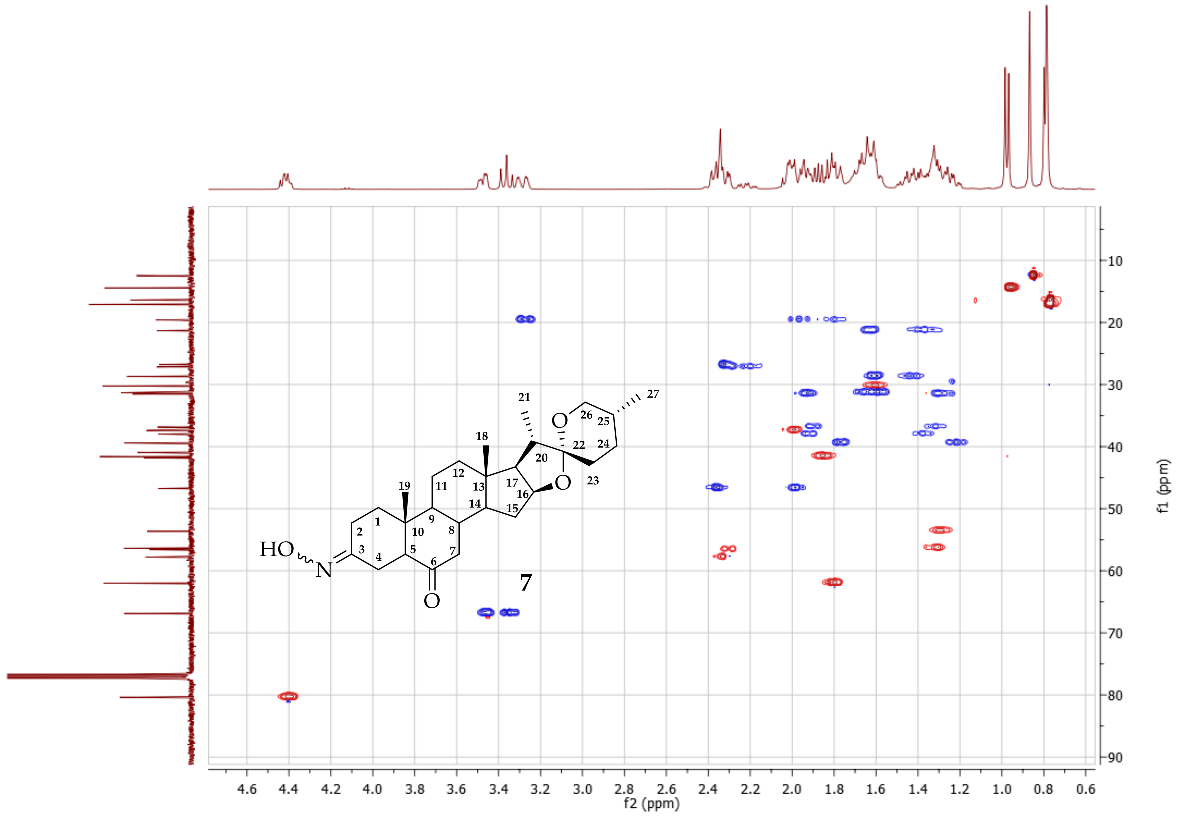

2D HMBC

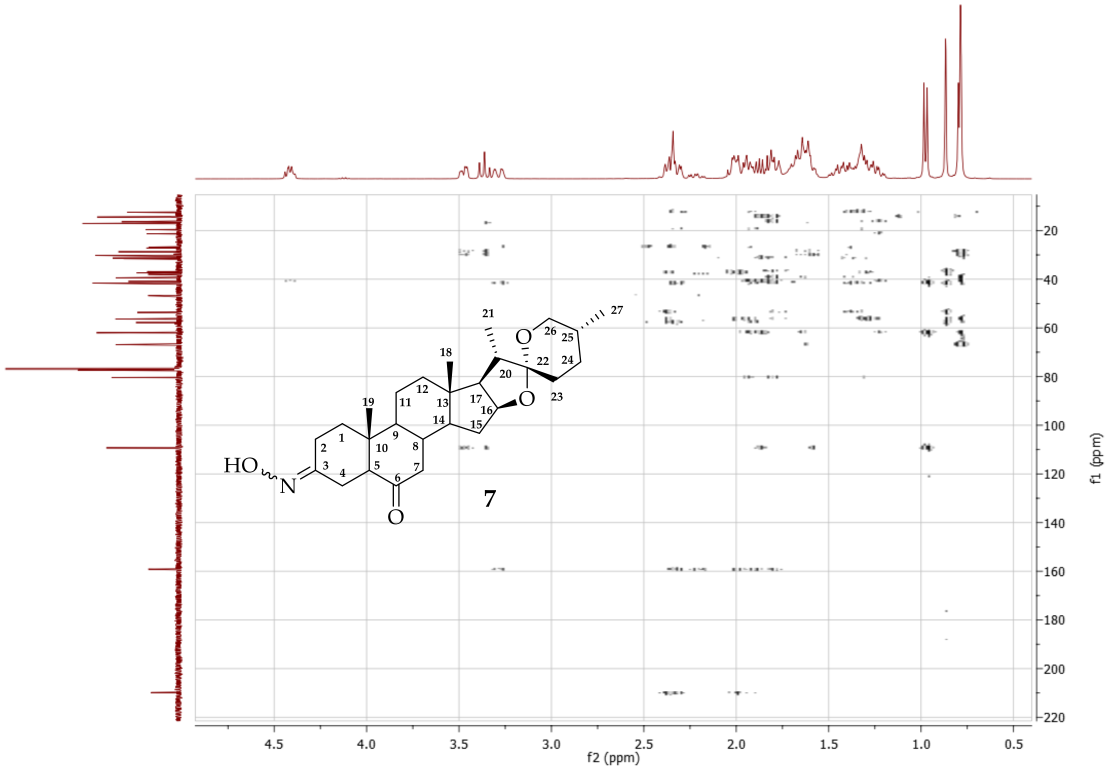

***Figure S8**: NMR spectra of mixture (25R)- (3E,6E)-dihydroximino-5α-spirostane (**8a**) and (25R)- (3Z,6E)-dihydroximino-5α-spirostane (**8b**)*

^{1}H-NMR

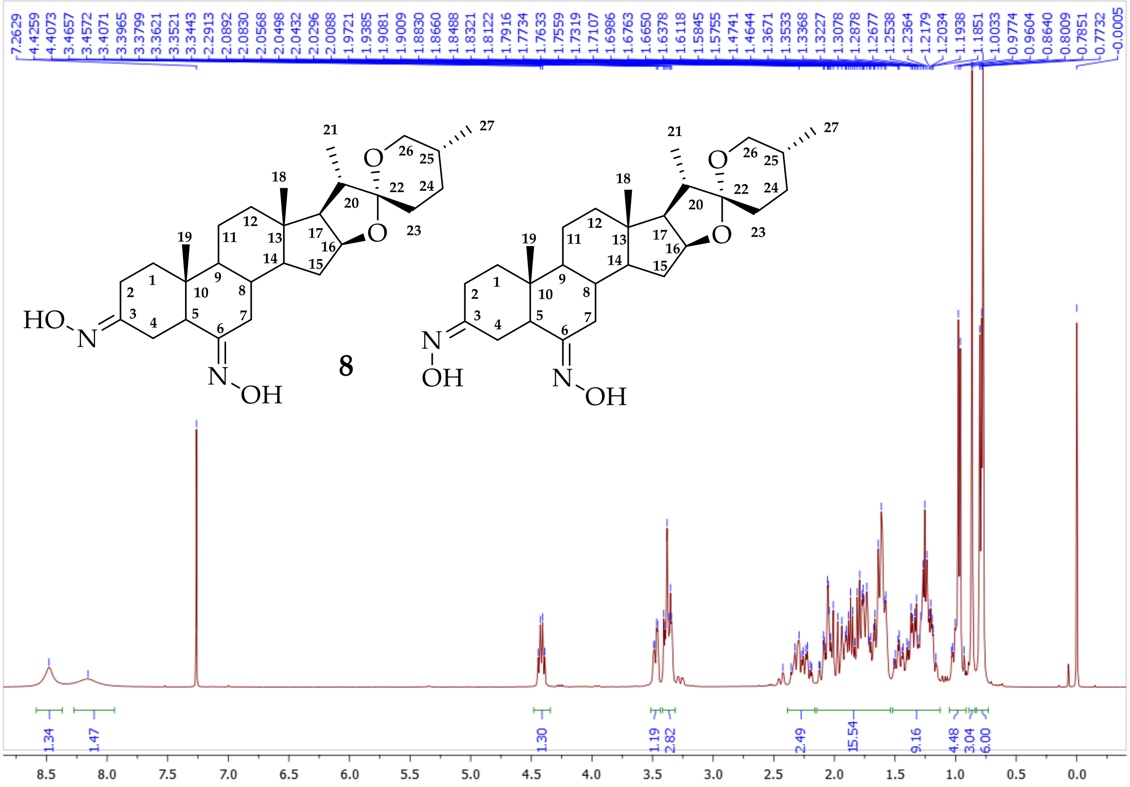

^{13}C-NMR

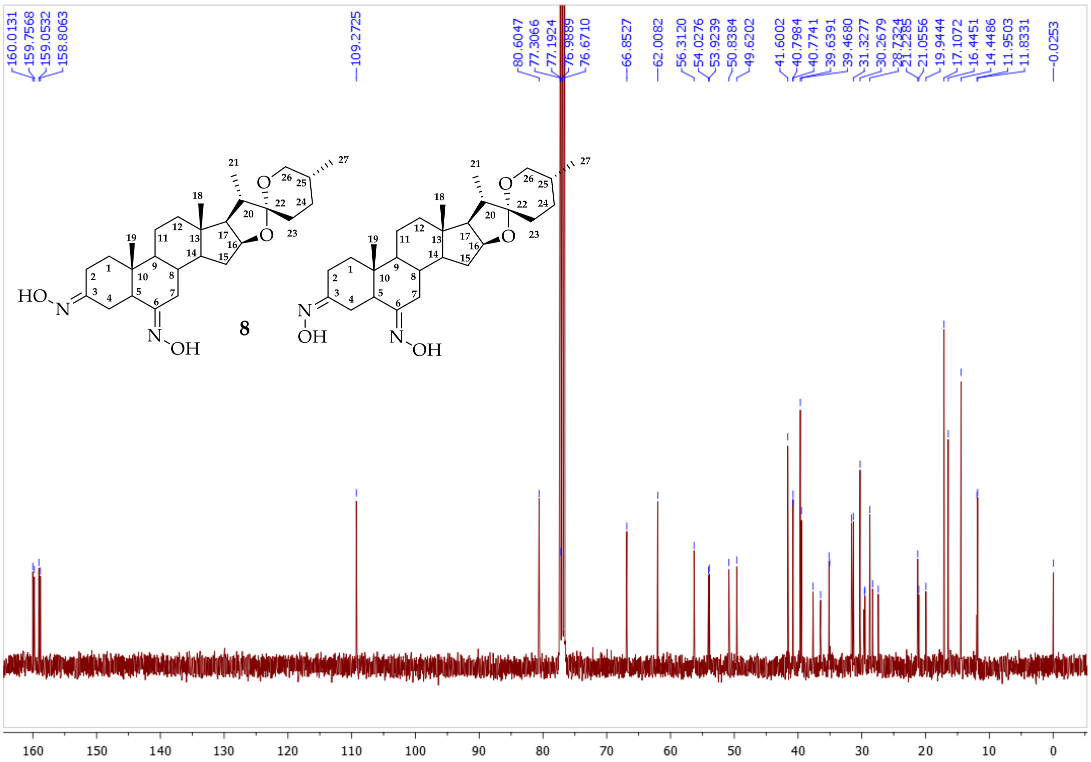

2D COSY

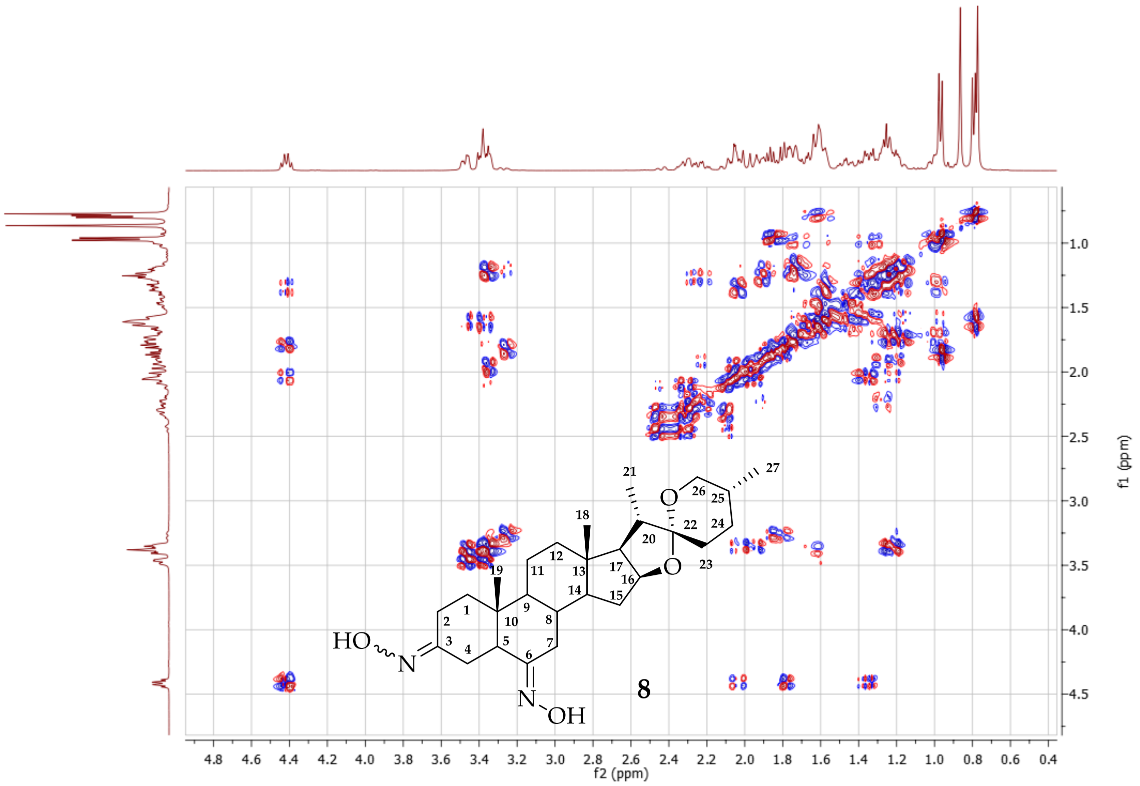

2D HSQC

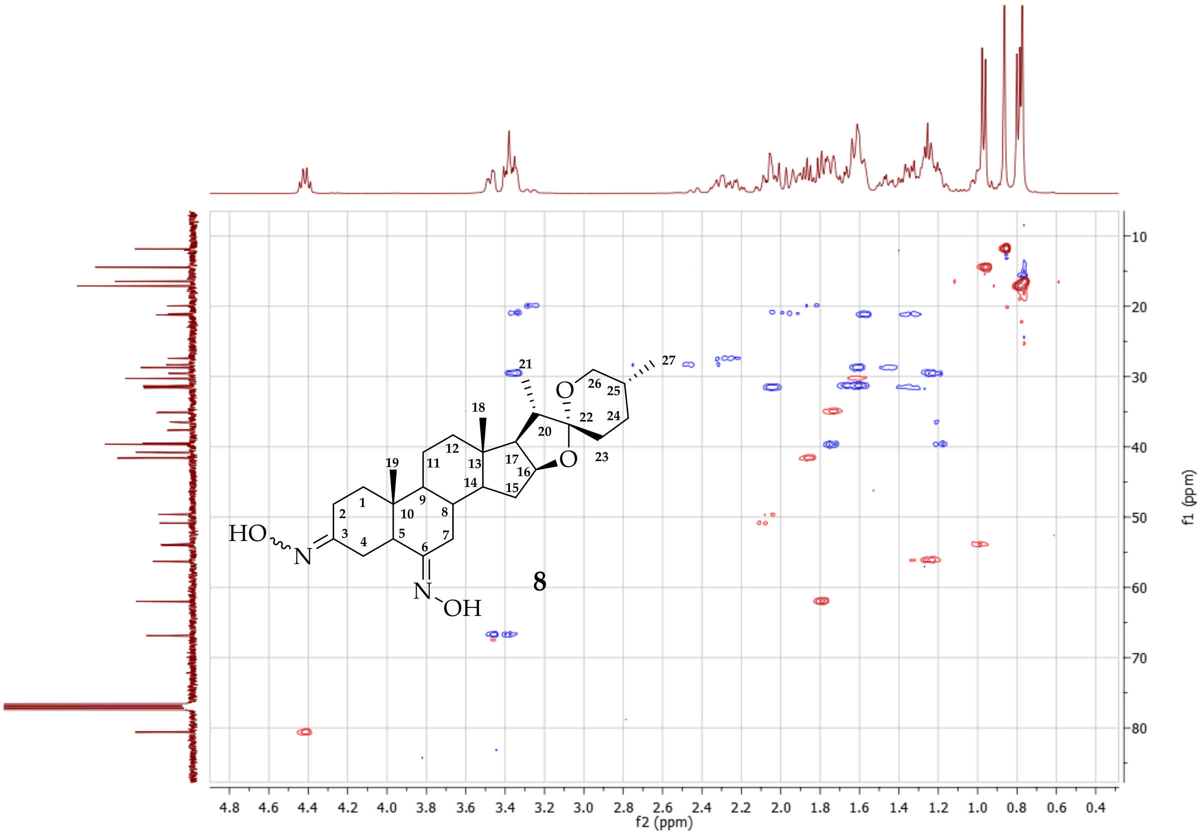

2D HMBC

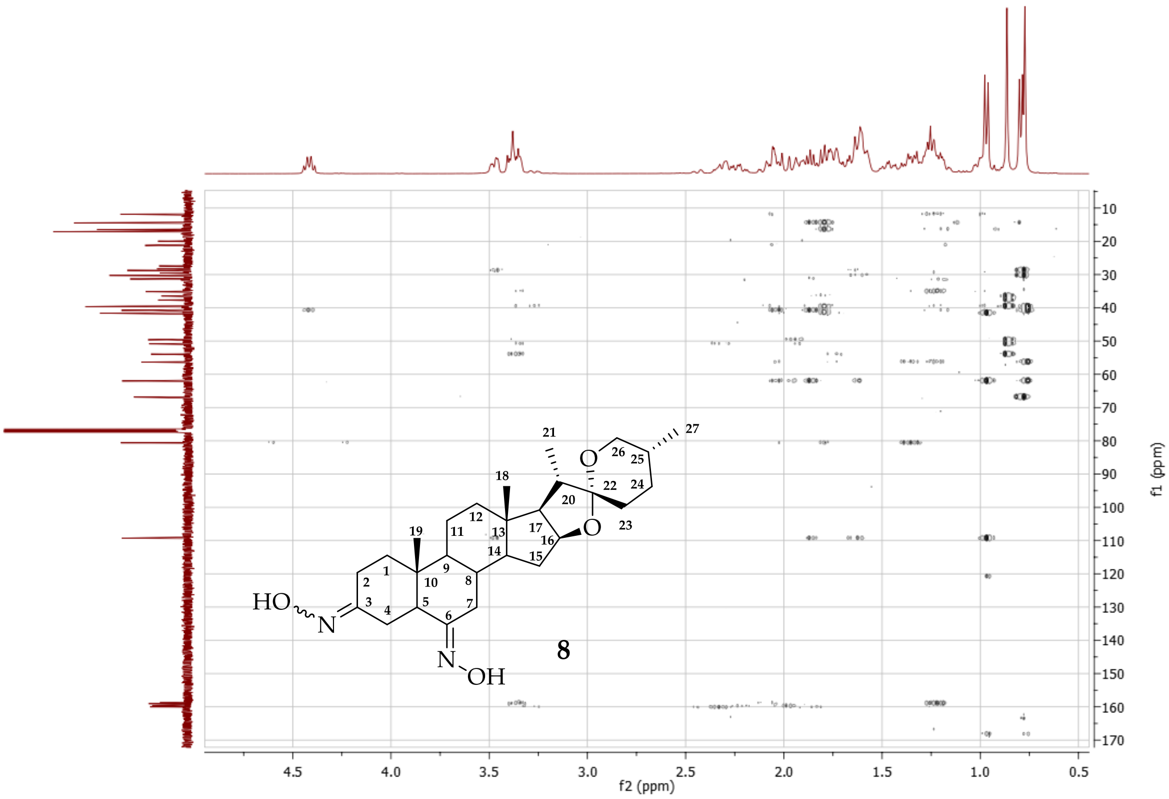

***Figure S9**: NMR spectra of (25R)-3β,5α-dihydroxy-spirostan-6-ona (**9**)*

^{1}H-NMR

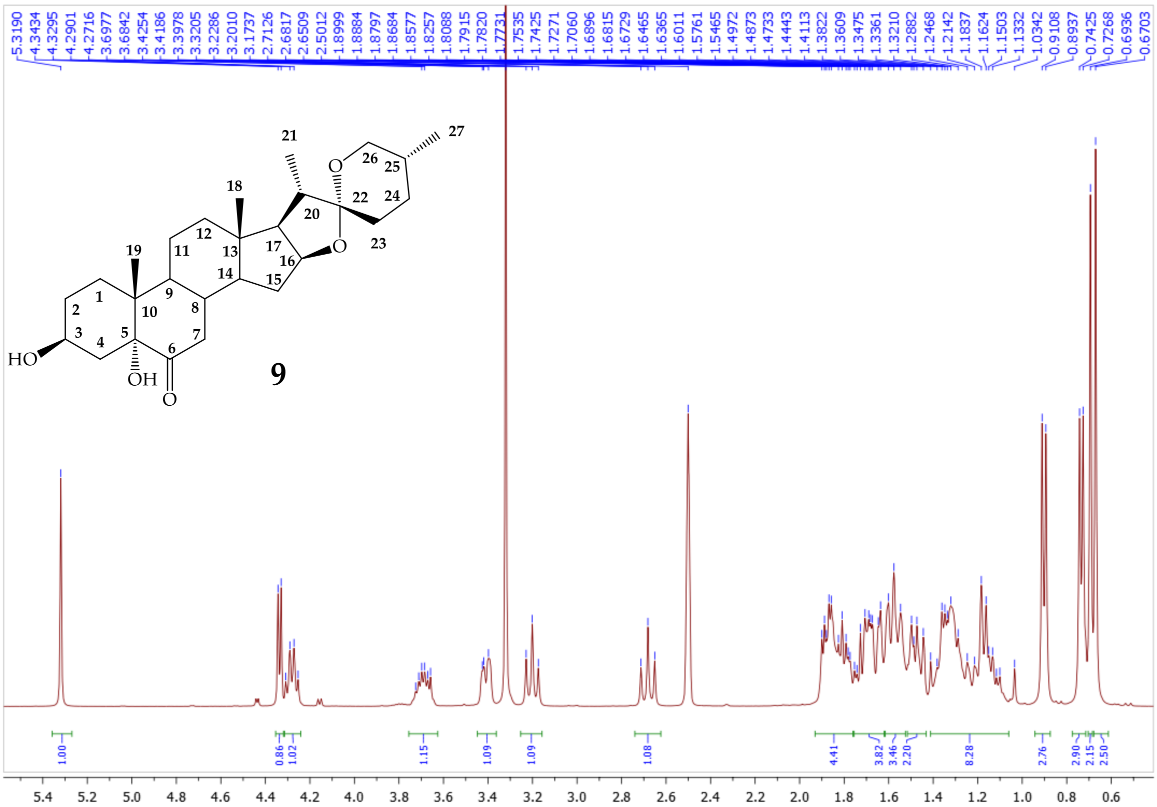

^{13}C-NMR

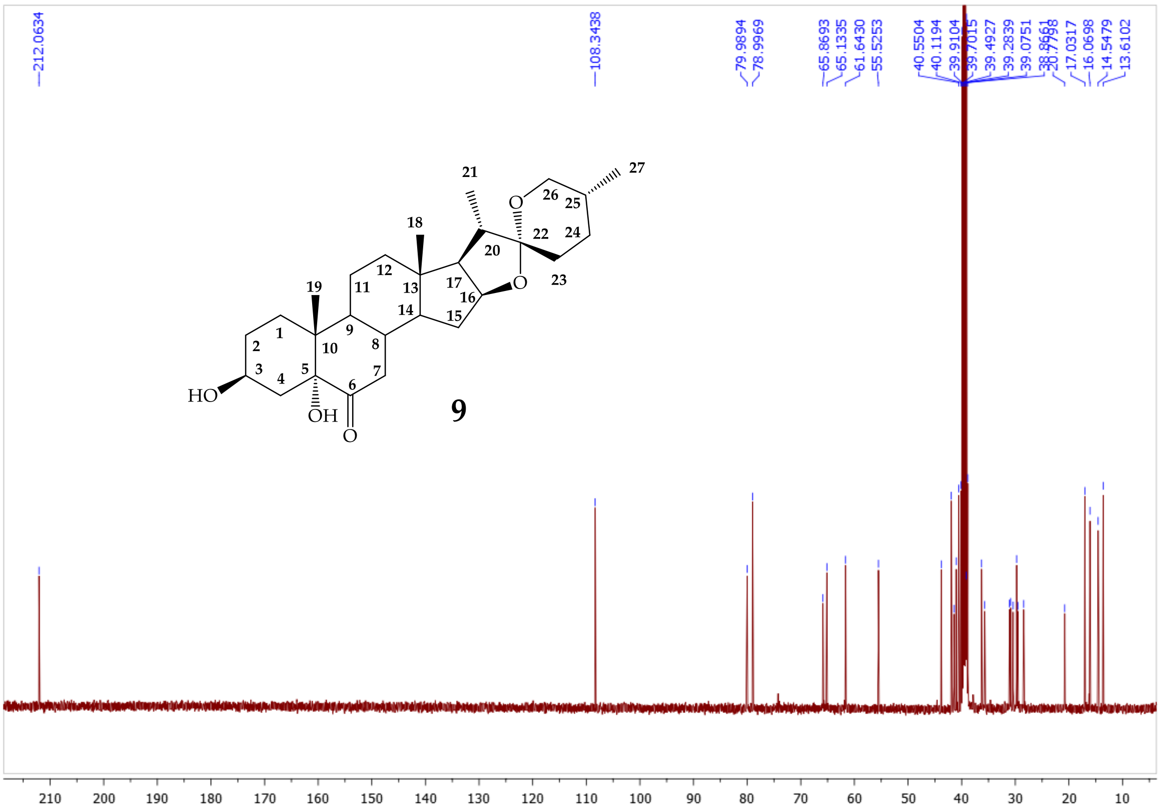

Figure S10: *NMR spectra of (25R)- (6E)-hydroximino-5α-spirost-3β,5-diol (**10**)*

^{1}H-NMR

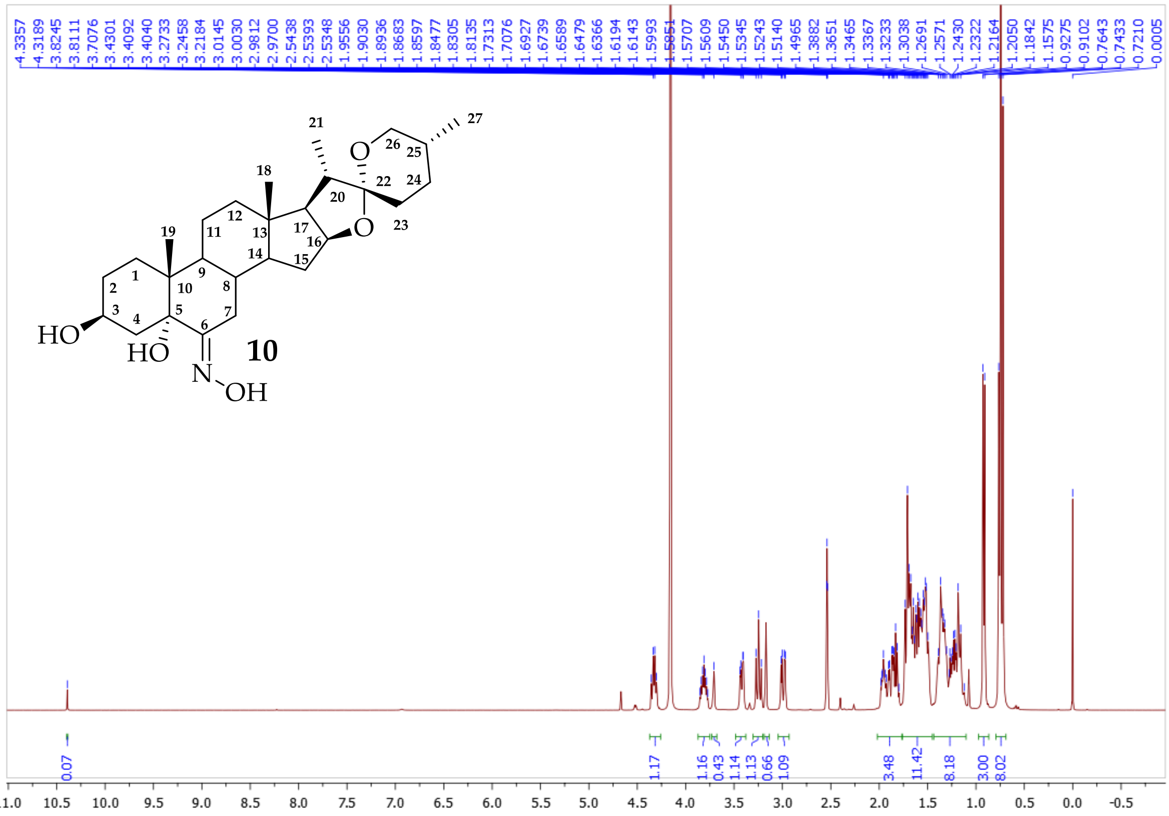

^{13}C-NMR

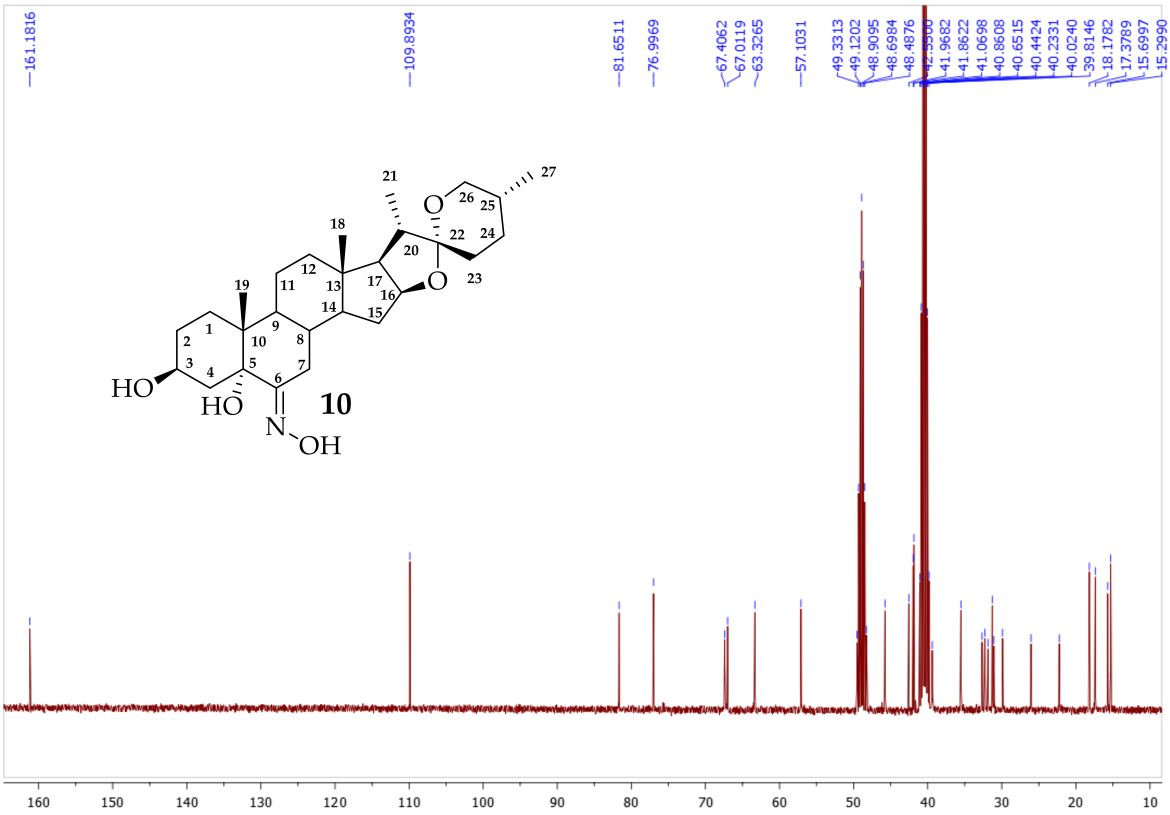

Figure S11: *NMR spectra of (25R)-5-oxo-5.6-secospirost-3-en-6-nitrile (**11**)*

^{1}H-NMR

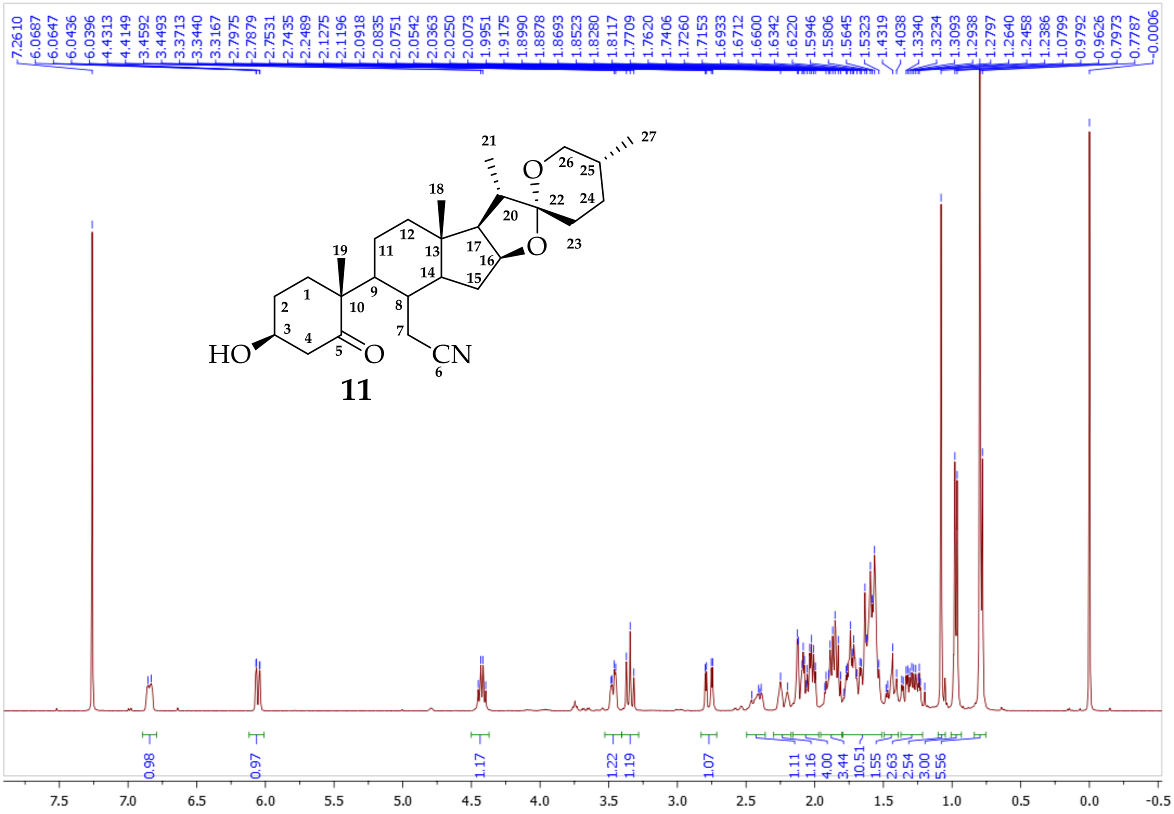

^{13}C-NMR

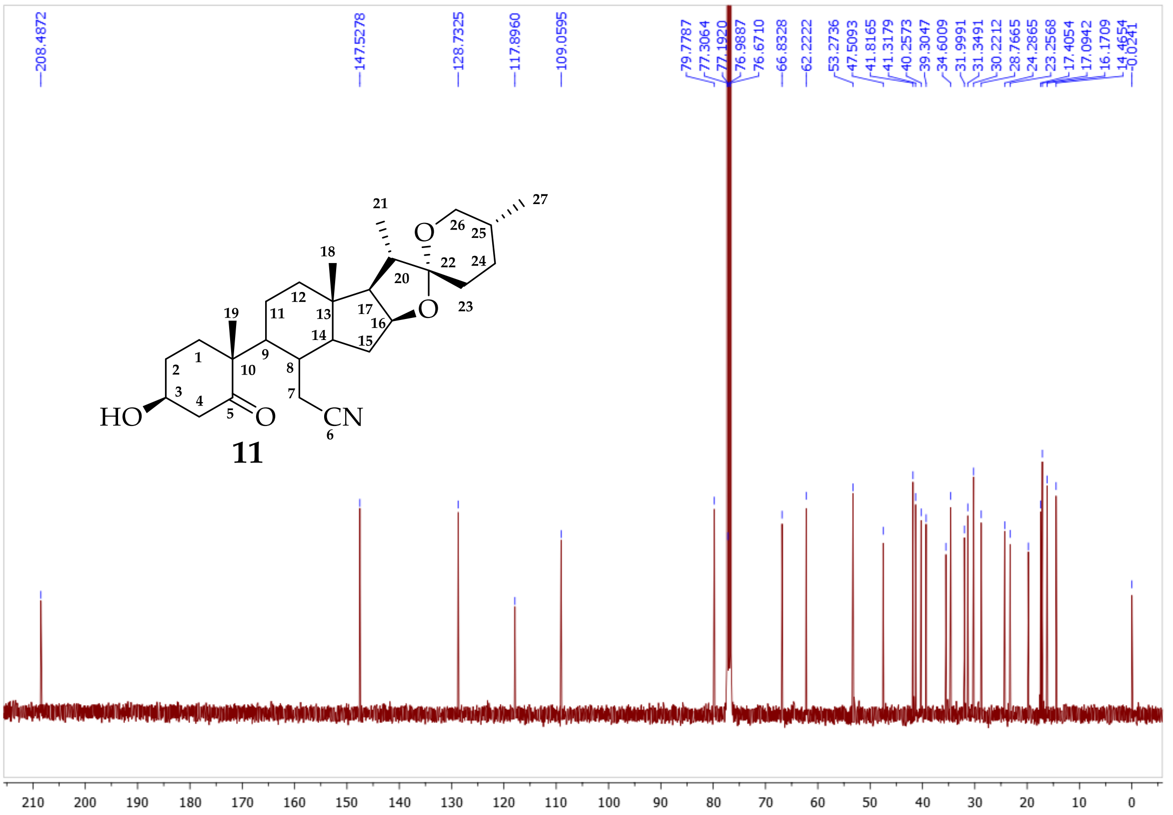

^{13}C-DEPT-135 NMR

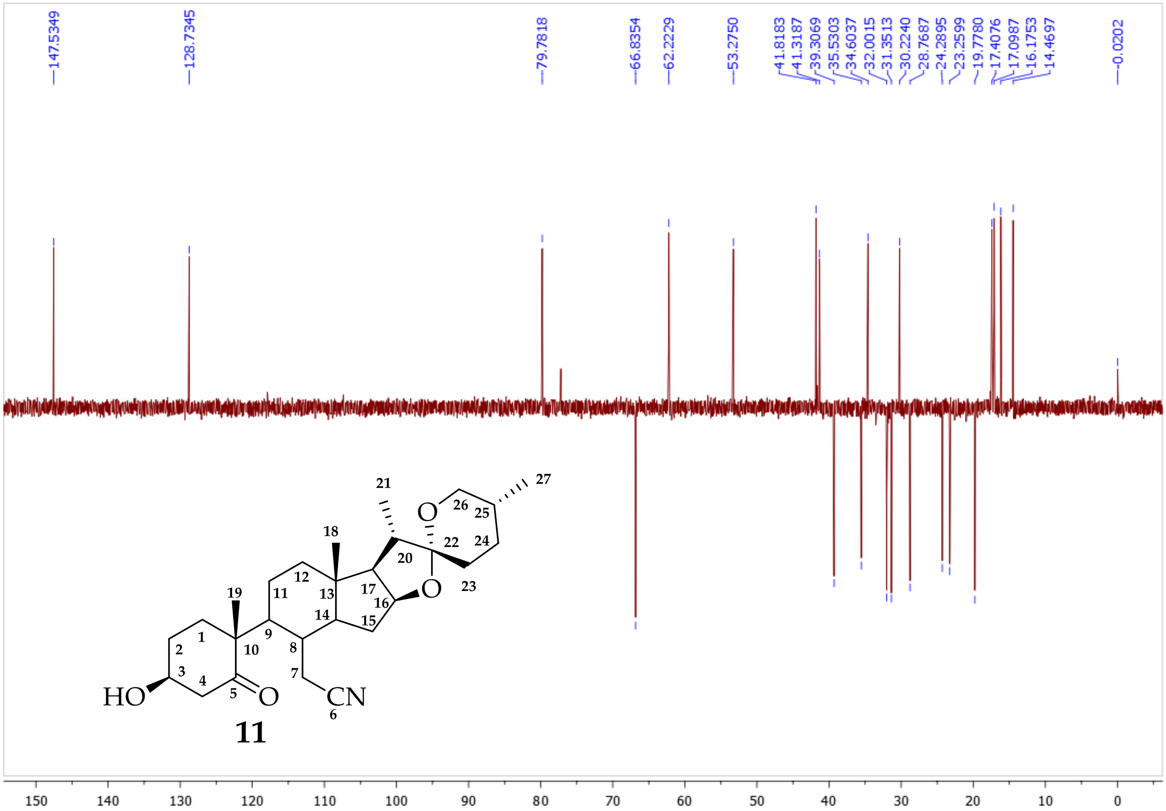

Disclaimer/Publisher's Note: The statements, opinions and data contained in all publications are solely those of the individual author(s) and contributor(s) and not of MDPI and/or the editor(s). MDPI and/or the editor(s) disclaim responsibility for any injury to people or property resulting from any ideas, methods, instructions or products referred to in the content.
